# Exosomal SLC16A1-AS1-induced M2 macrophages polarization facilitates hepatocellular carcinoma progression

**DOI:** 10.7150/ijbs.94440

**Published:** 2024-08-12

**Authors:** Yuhang Hu, Yang Li, Hewei Xiong, Ya Zhang, Fan Wang, Wenfeng Zhuo, Zhu Zeng, Yong Zhao, Hongda Wang, Ping Hu, Shengbo Han, Yan Huang, Guozheng Lv, Gang Zhao

**Affiliations:** Department of Emergency Surgery, Union Hospital, Tongji Medical College, Huazhong University of Science and Technology, Wuhan 430022, China.

**Keywords:** exosomes, SLC16A1-AS1, M2 polarization, macrophages, m^6^A

## Abstract

Macrophages are the most abundant alternative immune cells in the tumor microenvironment (TME). The cross-talk between macrophages and tumor cells provides an important shelter for the occurrence and development of tumors. As an important information transfer medium, exosomes play an important role in intercellular communication. Nonetheless, how exosomal lncRNAs coordinate the communication between tumor cells and immune cells in hepatocellular carcinoma (HCC) is incompletely understood. We found that HCC exosomes-derived antisense RNA of SLC16A1(SLC16A1-AS1) promoted the malignant progression of HCC by regulating macrophage M2-type polarization. Mechanistically, the HCC exosomal SLC16A1-AS1 enhanced mRNA stabilization of SLC16A1 in macrophage by promoting the interaction between 3' untranslated regions (3'UTR) of SLC16A1 mRNA and heterogeneous nuclear ribonucleoprotein A1 (HNRNPA1). As a lactate transporter, SLC16A1 accelerated lactate influx and then activated c-Raf/ERK signaling to induce M2 polarization of macrophages. Reciprocally, M2 macrophages secreted IL-6 to activate STAT3 and then induce METTL3 transcription in HCC cells, which increasing m6A methylation and stabilization of SLC16A1-AS1. In turn, the reciprocal SLC16A1-AS1/IL-6 signaling between HCC cells and M2 macrophages promoted the proliferation, invasion and glycolysis of HCC cells. Our study highlights that exosomal SLC16A1-AS1 acts as a signaling message that induces lactate-mediated M2 polarization of macrophages, and implies that SLC16A1-AS1 might be an applicable target for therapeutic treatment of HCC.

## 1. Introduction

Liver cancer is the sixth most common malignancy in the world and the third most common cause of cancer death. Hepatocellular carcinoma (HCC) accounts for 75%-85% of primary liver cancer cases. Despite advances in prevention, screening, and new diagnostic and treatment techniques, treatment for HCC has hit a bottleneck, the five-year survival rate (<15%) for advanced HCC is dismal 1, which suggests that HCC is still a highly fatal disease. Therefore, the discovery of new diagnostic biomarkers and a better understanding of molecular mechanisms underlying HCC progression and metastasis are urgently needed, which will contribute to developing therapeutic options and extending the survival time for HCC patients.

The tumor microenvironment (TME) is necessary for tumor cell survival and plays an indispensable role in the development and progression of cancer. Studies have shown that the tumor microenvironment plays an essential role in tumor growth 2, infiltration metastasis 2, apoptosis 3 and immune escape 4. The tumor microenvironment comprises diverse non-malignant stromal cell types including tumor-associated macrophages (TAMs), the most abundant leukocyte infiltrates. Numerous studies have shown that the high infiltration of TAMs is associated with poor clinical outcomes in several kinds of solid cancers, including bladder, gastric cancer, and hepatocellular carcinoma 5-7. Macrophages exhibit different phenotypes and functions in response to various microenvironmental signals generated from tumor and stromal cells 8. In response to microenvironmental signals, inactivated macrophages (M0) can be broadly divided into M1 (classically activated) like, and M2 (alternatively activated) like macrophages. The M1 macrophages have inflammation-promoting and anti-tumor effects, while M2 macrophages promote tumor angiogenesis, immune escape, migration, and metastasis of cancer 8. Notably, TAMs are phenotypically described as M2 macrophages. Numerous reports indicated that highly infiltrated M2 macrophages contributed to HCC progression and were closely associated with poor prognosis in HCC patients. Oscar W.H. et al. showed that M2 macrophages contributed to poor prognosis in HCC and promoted tumor invasiveness through CCL22-induced EMT 9. In a hypoxic-inflammatory microenvironment, M2 macrophages-derived IL-1β enhances EMT and progression of HCC 10. In addition, sorafenib inhibits the growth of hepatoma cells by interfering with the secretion of insulin-like growth factor-1(IGF-1) by M2 macrophages 11. Thereby, these results remind that M2 macrophages would be a valuable potential target for the treatment of HCC. Therefore, it is very important to explore the mechanism for M2 polarization of macrophages in HCC.

Recently, tumor-derived exosomes have played an increasingly important role in regulating macrophage polarization 12. Studies showed that hypoxic tumor-derived exosomes from human melanoma, skin, and lung cancer cell lines promoted macrophage M2 polarization by phosphorylation of STAT6 13. Tumor-derived exosomal miR-301a regulated M2 macrophage polarization through the PTEN/PI3K pathway, thus promoting the metastasis of pancreatic cancer 14. Ham et al. reported that breast cancer-derived exosomes induced M2 polarization of macrophage via gp130/STAT3 signaling 15. Similarly, exosomal miR-146a-5p induced M2-polarization of macrophages byactivation of STAT3 signaling, therby promoting the progress of HCC progression 16. Nevertheless, the mechanism behind the regulation of macrophage polarization by molecules in HCC-derived exosomes remains unclear, and more research is needed to explore the relationship between HCC-derived exosomes and macrophages.

Long noncoding RNAs (lncRNAs) are a class of noncoding transcripts of more than 200 nucleotides with limited protein-coding capacity 17, which can regulate gene expression at various levels, including epigenetic gene regulation, transcriptional and posttranscriptional processing 18. LncRNAs play essential roles in regulating tumorigenesis 19, metabolism 20, and drug resistance 21. Recently reports have suggested that lncRNAs can regulate the tumor microenvironment remodeling and macrophage polarization. LncRNA-BCRT1 promoted M2 polarization of macrophages of breast cancer by targeting miR-1303/PTBP3 axis 22. LncRNA-MM2P modulated M2 macrophages polarization by phosphorylating STAT6, thus promoting tumor angiogenesis and tumorigenesis 23. LINC00662 induces M2 macrophages polarization in HCC via activating Wnt/β-catenin signaling 24. Furthermore, research also demonstrated that exosomal lncRNA could induce M2 polarization of macrophages in HCC. Such as, data from Li et al. showed HCC-derived exosomal lncRNA TUC339 induces macrophage M2 polarization 25. Moreover, lncRNA HMMR-AS1 in HCC exosomes competed with miR-147 to prevent the degradation of ARID3A, thereby promoting M2 polarization of macrophages 26. Although studies have indicated the pivotal role of HCC exosomal lncRNAs in regulating M2 macrophage polarization, the detailed mechanisms have not been thoroughly dissected yet.

In this study, we first investigated whether HCC cell-derived exosomes could induce M2 polarization of macrophage. Furthermore, we analyzed the expression of exosomal lncRNAs by RNA-seq and found that antisense RNA of SLC16A1 (SLC16A1-AS1) was significantly enriched in the exosomes derived from various HCC cell lines. We further evaluated the effects of HCC exosomal SLC16A1-AS1 on the M2 polarization of macrophages. Functional experiments were continually performed to explore the mechanism for exosomal SLC16A1-AS1 inducing M2 polarization of macrophages through inducing lactate influx. Meanwhile, we further explored the reciprocal function of M2 macrophages on SLC16A1-AS1 expression and glycolysis in HCC cells. Our results provide novel insight into the polarization mechanism of macrophages in the microenvironment of HCC and a promising therapeutic target for HCC.

## Materials and Methods

### Clinical samples

We collected a total of 92 pairs of HCC tissue samples and their corresponding adjacent non-tumorous tissue samples, along with relevant clinical information, from patients who underwent hepatectomy at the Department of Hepatology Surgery in Union Hospital (Wuhan, China). Histopathological diagnosis was performed by two pathologists following the guidelines set by the National Comprehensive Cancer Network (NCCN). A portion of the excised tissue specimens was fixed in 10% buffered formalin solution and embedded in paraffin, while another portion was immediately frozen using liquid nitrogen after surgical resection. Signed informed consents were obtained from all participants, and this study received approval from the Ethics Committee of the Academic Medical Center at Huazhong University of Science and Technology. All procedures conducted during this study adhered to the principles outlined in the Declaration of Helsinki.

### Exosome isolation and identification

HepG2/MHCC97H/MIHA Cells (1 × 10^6^/well) were plated in a vesicle-depleted medium for 2 days prior to the collection of exosomes. The medium was first centrifuged at 300× g for 10 min and then at 2000× g for 20 min at 4 °C to remove cells and then at 10,000× g for 20 minutes at 4 °C. The supernatant was then centrifuged at 100,000× g for 70 min at 4 ◦C to pellet exosomes, which were then washed by resuspending in PBS and ultra-centrifuged at 100,000× g for 70 min 4 °C. The final pellet was re-suspended with 50 to 100 ul PBS and stored at 4 °C or -80 °C for subsequent experiments.

The extracted exosomes were examined by electron microscopy using negative staining. Furthermore, the size and quantitation of exosomes were analyzed using a NanoSight NS300 instrument (Malvern Instruments) equipped with NTA 3.0 analytical software (Malvern Instruments).

### Exosome labeling and tracking

Purified exosomes isolated from the culture medium were collected and labeled with PKH26 Red Fluorescent membrane linker dye (Sigma-Aldrich) according to the manufacturer's instructions. Then, labeled exosome pellets were resuspended and added to the unstained macrophages for exosomes uptake studies. After incubation for 30 minutes, 2 hours, or 12 hours at 37 °C, cells were observed by fluorescence microscopy.

### RNA sequencing assay

Total RNA was isolated with Trizol from exosomes derived from two HCC cell lines (HepG2 and MHCC97H) and MIHA cell. RNA purity was assessed using the ND-1000 Nanodrop. RNA integrity was evaluated using the Agilent 2200 TapeStation (Agilent Technologies, USA) and each sample had the RIN above 7.0. In brief, rRNAs were removed from total RNA using EpicentreRibo-Zero rRNA Removal Kit (Illumina, USA) and then fragmented to approximately 200bp. Next, the purified RNAs were subjected to first-strand and second-strand cDNA synthesis followed by adaptor ligation and enrichment with a low-cycle according to instructions of NEBNext® Ultra™ RNA Library Prep Kit for Illumina (NEB, USA). The purified library products were evaluated using the Agilent 2200 TapeStation and Qubit®2.0 (Life Technologies, USA). The libraries were paired-end sequenced (PE150, Sequencing reads were 150 bp) at Guangzhou RiboBio Co., Ltd. (Guangzhou, China) using the IlluminaHiSeq 3000 platform.

### RNA immunoprecipitation (RIP)

In order to detect RNA-protein binding complexes, an RIP assay was performed using a Magna RIP™ RNA binding protein immunoprecipitation kit (Magna RIP™, Millipore, USA) according to the manufacturer's instructions. Firstly, we use cell lysis buffer containing protease inhibitors and RNase inhibitors to obtain lysates. Secondly, magnetic beads were pre-incubated with an anti-HNRNPA1 antibody (1:100, Cell Signaling Technology, 8443), anti-IGF2BP3 antibody (1:20, Proteintech, 14642-1-AP), negative control IgG respectively for 30 minutes at room temperature, and then lysates were immunoprecipitated with magnetic beads bound antibody for 3 hours to overnight at 4°C. Finally, the immunoprecipitate was washed withPBS 5 times to remove unbound materials, and then the RNA-protein complexes were treated with proteinase K, the immunoprecipitated RNAs were purified and analyzed by qPCR. RNA levels were normalized to the input.

### Biotin-RNA pull-down assay and mass spectrometry analysis

The full length or truncates of SLC16A1-AS1 and SLC16A1 sequence were amplified by PCR with T7-containing primer and then transcribed by MAXIscript™ T7 Transcription Kit (Thermo Fisher Scientific, MA, USA) according to the manufacturer's instructions. The newly synthesized RNA was Biotin-labeled with Pierce™ RNA 3' End Desthiobiotinylation Kit (Thermo Fisher Scientific, MA, USA). HepG2 cells were collected to obtain cell lysate. RNA pull-down assays were performed with a Pierce Magnetic RNA-Protein Pull-Down Kit (Thermo Scientific, USA). According to the manufacturer's instructions, the biotin-labeled RNA was captured by Streptavidin-coupled Dynabeads (Invitrogen, USA) and incubated with cell lysates at 4 °C for 6 hours to overnight, then the RBP complex was washed and eluted. Retrieved protein was detected by Western blot or mass spectrometry analyses at Shanghai Applied Protein Technology Co. Ltd. Primers for pull-down were indicated in Supplementary Table S4.

### ^14^C-Lactate uptake assay

Lactate uptake by macrophages was examined using uniformly labeled ^14^C-lactate. Cells seeded in 12-well plates were equilibrated in 250 µL 10 mM HEPES (pH 7.50), 5 mM KCl, 100 mM NaCl, 1 mM MgCl2 (uptake buffer) containing 2 µCi (100 μM; Amersham Biosciences) ^14^C-lactate and uptake stopped after 2 h incubation at 37 °C. Uptake was stopped by washing four times with ice-cold PBS. Cells were lysed with 250 μL of 0.1 M NaOH. The radioactivity was determined by mixing 750 μL of scintillation liquid with 200 μL of cell lysate and counted with a liquid scintillation counter.

### Animal experiments

The animal experiments reported in this study were approved by the Animal Research Committee of the Academic Medical Center at Huazhong University of Science and Technology. All procedures conformed to the guidelines of the Institutional Animal Care and Use Committees, with careful consideration for the humane treatment of the animals. For the subcutaneous tumor growth assay, 6-8 weeks old nude mice were sorted into six groups (n = 5 per group) at random. Each group received one of the following treatments bilaterally into the subcutaneous tissue of the flank: MHCC97H cells alone (1×10^6), exosomes from M0 macrophages incubated with MHCC97H cells (1×10^6), MHCC97H with AS1-KD exosome-treated M0 macrophages (1×10^6), and MHCC97H coupled with exosomes from siNC treated M2 macrophages (1×10^6) or siIL-6 treated M2 macrophages (1×10^6). Tumor volumes were measured every 4 days by the formula V = 0.5 × length × width^2, and the observation endpoint for the subcutaneous tumor size was established when tumors reached a maximum diameter of 2cm. After monitoring for 28 days, under general anesthesia, all mice were humanely sacrificed, and tumors were extracted for examination. Regarding the lung metastasis experiment, groups of cells in 100 μl were injected via the tail vein. The observational endpoints considered were clinical endpoints such as significant weight loss and reduced mobility, in addition to the standard methodologies of monitoring. Thirty days after the injection, upon reaching any of these predefined clinical endpoints that signify a humane endpoint as well, or at the end of the study period, mice were euthanized, and metastatic burden was examined via necropsy, complemented by histological investigation of tumor, liver, and lung tissues using H&E and IHC staining.

### Statistical analyses

SPSS v22.0 and GraphPad Prism 8 were used for analyses, and all results were at least three independent experiments and were presented as means ± standard deviation (SD). Differences between groups were analyzed using *t*-tests and Chi-squared tests. The Pearson correlation coefficient was used to analyze the correlation between two genes. Kaplan-Meier analysis and a log-rank test were used to compare the different survival rates. All data were analyzed using two-tailed tests. The difference was regarded to be significant at * p<0.05, ** p<0.01, *** p<0.001. N.S. indicates non-significance.

### Supplementary Methods

Cell culture, Exosomes Co-cultured with macrophages, Quantitative real-time PCR (qRT-PCR), Western blot analysis, Measurement of Glucose and Lactate, Colony Formation Assay, Cell transwell invasion assays, Transfection, Chromatin immunoprecipitation (ChIP) assay, Quantification of cytokines by enzyme-linked immunosorbent assay (ELISA), RNA- Fluorescence in situ hybridization (RNA- FISH), Immunohistochemistry (IHC), Immunofluorescence (IF), RNA stability, Cytoplasmic and nuclear RNA isolation, Luciferase reporter assay, MeRIP, Flow cytometry are given in Supplementary Methods.

## Results

### HCC cells derived-exosomes promote M2 polarization of macrophages

**Figure 1:**
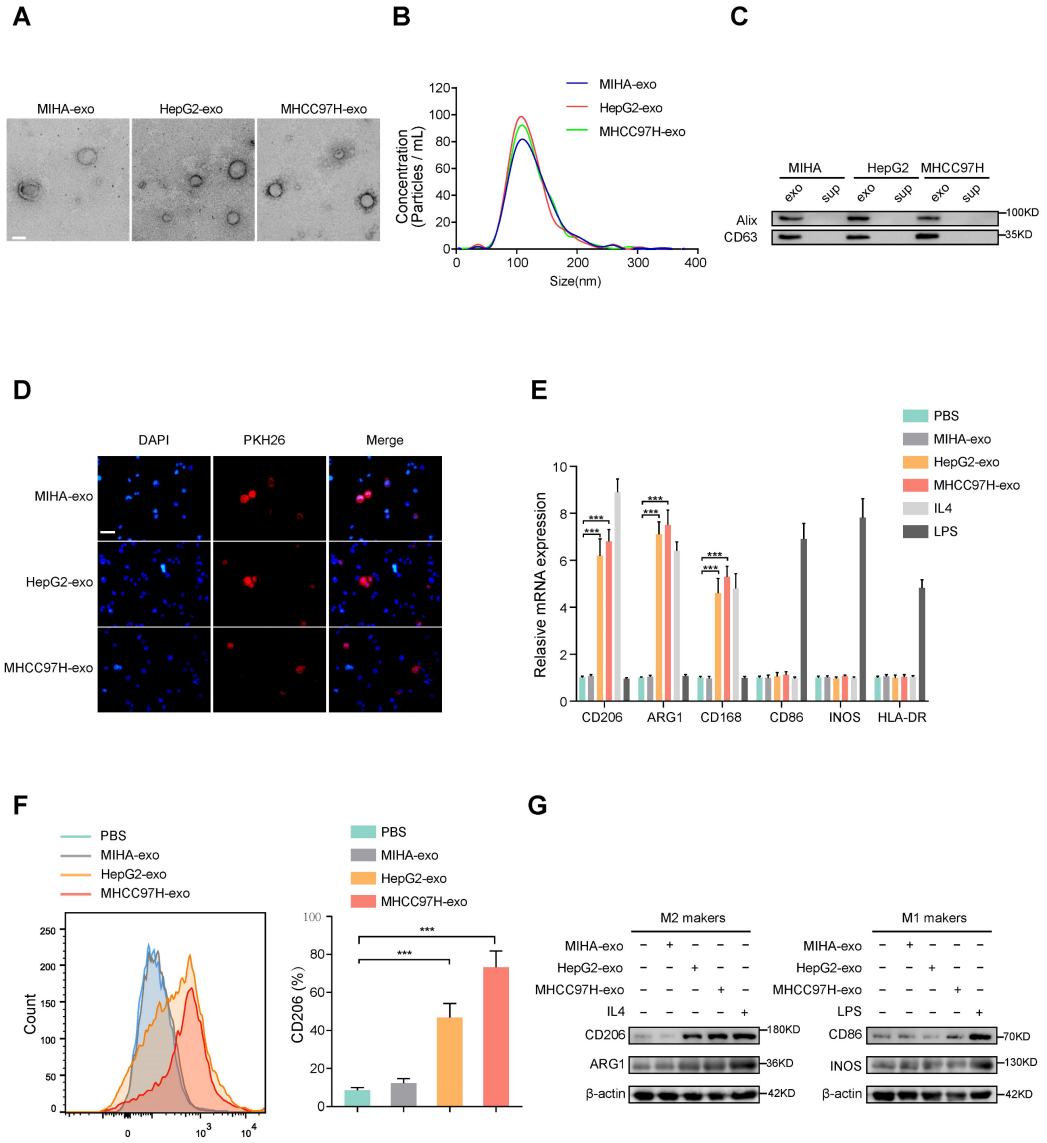
** HCC cells-derived exosomes promote M2 polarization of macrophages. (A)** Transmission electron micrograph of HCC cell-derived exosomes and human normal liver cell line (MIHA)-derived exosomes. Scale bar: 200 nm. **(B)** Exosomes released by different HCC cells or MIHA cells were detected by NanoSight particle tracking analysis. **(C)** Exosome markers Alix and CD63 proteins were detected by western blot assay. **(D)** Internalization of PKH26-labeled exosomes (red) by macrophages examined by laser scanning confocal microscope. Scale bar: 50 μm. **(E)** Relative gene expression of M1 markers (CD86, INOS, HLA-DR) and M2 markers (CD206, ARG1, CD163) of macrophages treated with MHCC97H-exo, MIHA-exo (100 mg/mL), or control (PBS, LPS and IL4). **(F)** Flow cytometry was used to detect the expression of M2 marker (CD206) in macrophages treated with MIHA-exo, HepG2-exo, MHCC97H-exo, (100 mg/mL), or PBS. **(G)** Western blot analysis was used to detect M2 markers (CD206, ARG1) and M1 markers (CD86, INOS).

To examine whether HCC-derived exosomes can regulate macrophages polarization, exosomes were extracted from normal liver cells (MIHA) and HCC cells (HepG2 and MHCC97H cells). The morphology and diameter of the exosomes were evaluated by electron microscopy (Fig. 1A), and NanoSight analysis (Fig. 1B) respectively. Western blot analysis of proteins extracted from exosomes further confirmed the presence of the exosomal proteins Alix and CD63 (Fig. 1C). Human THP-1 monocytes were induced to differentiate into macrophages by incubation with phorbol 12-myristate 13-acetate (PMA), which caused obviously visible changes in the adherent morphology and the recognized macrophage markers CD68 and CD14 (Supplementary Fig. S1A, B). To prove whether exosomes could be taken up by macrophages, PKH26-labeled exosomes were co-cultured with unstained macrophages. As shown in Fig. 1D, exosomes were eventually internalized by macrophages. The expression of M2 markers (CD206, ARG1, CD163) obviously increased in macrophages incubated with IL-4 or exosomes derived from HepG2 and MHCC97H cells, but not with exosomes derived from MIHA cells (Figure 1E-G). However, the M1 markers (CD86, iNOS, HLA-DR) of macrophages were significantly induced after incubation with LPS, but not with exosomes derived from HCC cells or MIHA cells (Fig. 1E-G). Moreover, through bioinformatics analysis of immune cell infiltration in the TCGA liver cancer database, we found that M2 type macrophages accounted for the largest proportion (Supplementary Fig. S1C-D). Subsequent survival analysis showed that the survival time of HCC patients with high infiltration of total macrophages or M2 macrophages was significantly shortened, while the degree of infiltration of M1 macrophages had no effect on the survival time of HCC patients (Supplementary Fig. S1E). Thus, our results indicated that HCC cell-derived exosomes promote macrophages toward M2-type polarization, which contributes to HCC progression.

### Exosomal SLC16A1-AS1 is critical for the HCC exosomes-induced M2 polarization of macrophages

**Figure 2:**
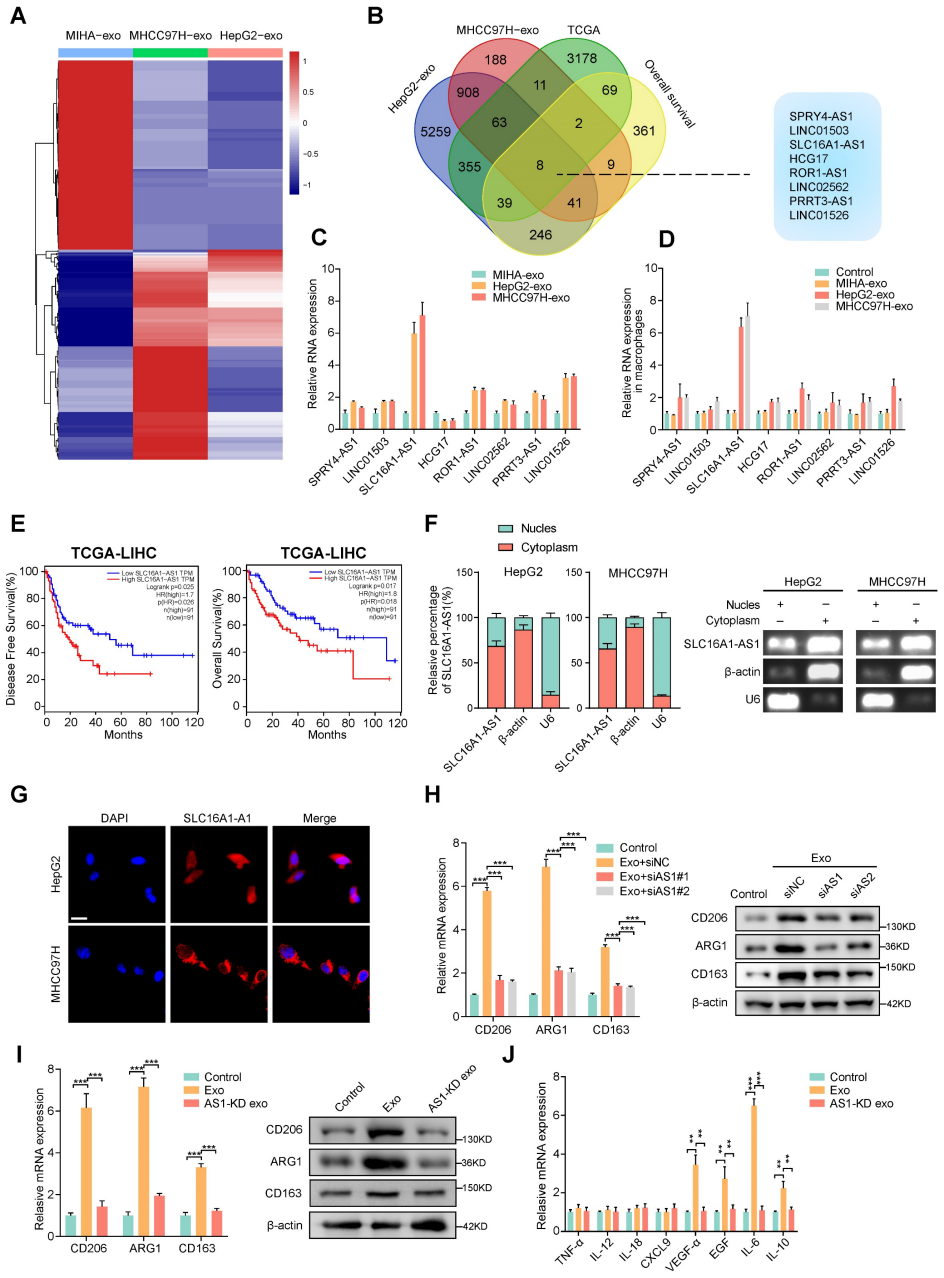
** Exosomal SLC16A1-AS1 is critical for the HCC exosomes-induced M2 polarization of macrophages. (A)** LncRNA sequencing of exosomal lncRNAs from different HCC cells (MHCC97H and HepG2 cells) and MIHA cells are presented in a heatmap. **(B)** Overlapping results of up-regulated lncRNAs in indicated groups. **(C)** Relative expression of lncRNAs in exosomes derived from HCC cells and MIHA cells. **(D)** Macrophages were incubated with HCC cells (MHCC97H and HepG2 cells)-derived exosomes or MIHA-derived exosomes for 24h. LncRNAs expression levels in macrophages were determined by qRT-PCR. **(E)** Kaplan-Meier analyses of disease-free survival (DFS) and overall survival (OS) in HCC patients with low and high levels of SLC16A1-AS1 using the log-rank test. **(F)** The expression of SLC16AS1-AS1 in nuclear and cytoplasmic extracts of HCC cells was detected by qRT-PCR. The PCR products were separated by 2% agarose gel electrophoresis; U6 and b-actin were used as markers of the nucleus and cytoplasm, respectively. **(G)** Fluorescence in situ hybridization (FISH) analysis showed the location of SLC16A1-AS1 in HCC cells. Blue: DAPI nuclear counterstaining. Scale bar: 20 μm. **(H)** Relative expression of M2 markers (CD206, ARG1, CD163) in macrophages after incubation with MHCC97H-derived exosomes alone or combined with SLC16A1-AS1 knockdown. **(I)** Relative expression of M2 markers in macrophages after incubation with MHCC97H-derived exosomes or SLC16A1-AS1 knockdown exosomes. **(J)** Relative macrophage-associated cytokines expression after incubation with exosomes or SLC16AS1 knockdown exosomes.

According to reports, exosomes contain a variety of biologically active molecules, including lncRNAs, which play important roles in regulating macrophages 22. In an attempt to identify specific lncRNA required for M2 macrophage polarization in exosomes derived from HCC cells, we conducted three rounds of screening in turn. Firstly, we utilized high-throughput lncRNA sequencing to generate lncRNA expression profiles of exosomes derived from two HCC cell lines (HepG2 and MHCC97H cells) and MIHA (Fig. 2A). Next, we selected the lncRNAs that were up-regulated (fold change > 1.5, *p* < 0.05) in in all of the exosomes that were derived from HepG2 and MHCC97H cells to intersect with those that were up-regulated (fold change > 1.5, *p* < 0.05) in TCGA liver hepatocellular carcinoma (LIHC) database and related to prognosis (*p* < 0.05). On the basis of overlapping analysis of these lncRNAs, 8 lncRNAs (SPRY4-AS1, LINC01503, SLC16A1-AS1, HCG17, ROR1-AS1, LINC02562, PRRT3-AS1, LINC01526) were singled out as candidates (Fig. 2B). Furthermore, aforementioned lncRNAs were subjected to validation by qRT-PCR in HepG2 and MHCC97H cell or MIHA cell-derived exosomes and their corresponding cells. Results show that the expression of SLC16A1-AS1 both in cells and exosomes was upregulated more than 4-fold in HepG2 and MHCC97H compared to MIHA (Fig. 2C and Supplementary Fig. S2A). Meanwhile, after incubated with exosomes derived from HCC cells, SLC16A1-AS1 was found to be the most significantly increased in co-cultured macrophages (Fig. 2D). Meanwhile, after treatment with RNase A and Triton X-100, the expression of SLC16A1-AS1 in medium of HCC cells decreased significantly (Supplementary Fig. S2B), suggesting that extracellular SLC16A1-AS1 is mainly encapsulated in exosomes with membrane structure instead of being directly released. Furthermore, treatment of macrophages with RNA transcription inhibitor actinomycin D (ActD, 5 μg/mL) did not affect the level of SLC16A1-AS1 in macrophages incubated with exosomes derived from HCC cells (Supplementary Fig. S2C), which confirmed that HCC cells exosomes did not regulate endogenous SLC1A1-AS1 transcription, but directly transferred SLC16A1-AS1 into macrophages. Kaplan-Meier survival curves revealed that HCC patients with high SLC16A1-AS1 expression had poor disease-free survival (DFS) and overall survival (OS) (Fig. 2E). Subcellular localization analysis showed that SLC16A1-AS1 mainly located in the cytoplasm (Fig. 2F and G). Consistent with the results, the RNA FISH analysis demonstrated that SLC16A1-AS1 was principally distributed in the cytoplasm (Fig. 2H). Online lncRNA tool LncBOOK 2.0 (https://ngdc.cncb.ac.cn/lncbook/) and a comparative genomics tool PyhloCSF indicated that SLC16A1-AS1 possesses very weak protein-coding potential (Supplementary Fig. S2D, E). In addition, the secondary structure of SLC16A1-AS1 was shown by the RNA fold web server (Supplementary Fig. S2F).

Moreover, the HCC cell-derived exosome-induced M2 polarization of macrophages was obviously inhibited by knockdown of SLC16A1-AS1 with siRNA (Fig. 2I). On the contrary, the overexpression of SLC16A1-AS1 remarkably induced M2 polarization of macrophages (Supplementary Fig. S2G). Notably, exosomes derived from SLC16A1-AS1 knockdown (AS1-KD) HCC cells failed to induce M2 polarization and upregulation of M2-associated cytokines (Fig. 2J, K). Furthermore, we detected SLC16A1-AS1 expression in different HCC cells and corresponding exosomes by qRT-PCR (Supplementary Fig. S2H). Data from the CCLE database (https://portals.broadinstitute.org/ccle) showed the level of SLC16A1- A S1 in various HCC cell lines and human cancers cell lines (Supplementary Fig. S2I, J). Pan-cancer analysis based on the TCGA database (https://portal.gdc.cancer.gov/) showed that SLC16A1-AS1 exhibits a high expression pattern in multiple gastrointestinal carcinomas including LIHC, colon adenocarcinoma (COAD), cholangiocarcinoma (CHOL), and esophageal carcinoma (ESCA) (Supplementary Fig. S2K). Taken together, these results suggested that exosomal SLC16A1-AS1 of HCC is a critical inducer for the M2 polarization of macrophages.

### Exosomal SLC16A1-AS1 induces M2 polarization by increasing SLC16A1 expression in macrophages

**Figure 3:**
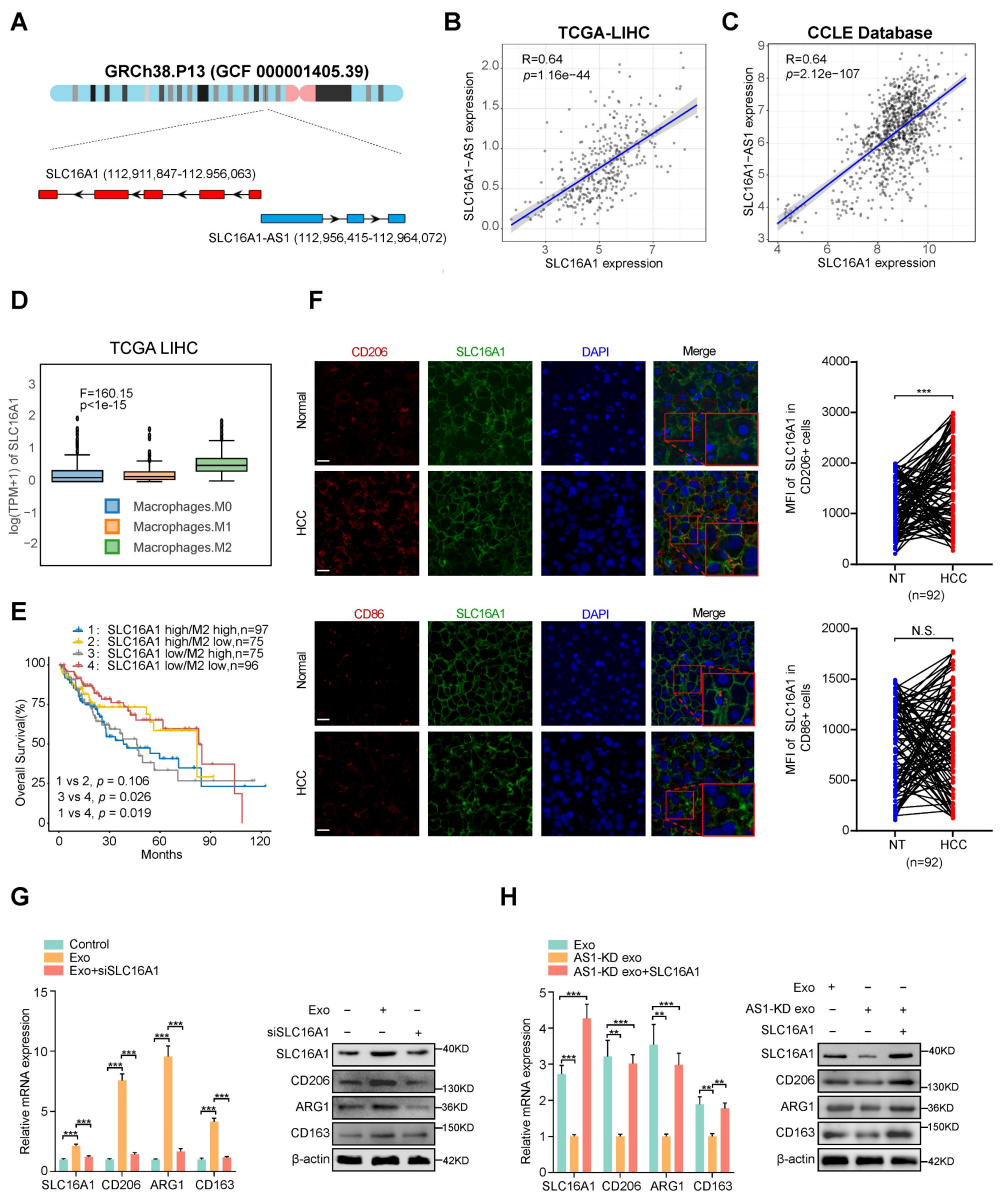
** Exosomal SLC16A1-AS1 induces M2 polarization by increasing SLC16A1 expression in macrophages. (A)** Schematic illustration showing the genomic location of SLC16A1-AS1 and SLC16A1. **(B)** Correlation analysis of SLC16A1-AS1 and SLC16A1 in HCC. **(C)** Correlation analysis of SLC16A1-AS1 and SLC16A1 in all cell lines from the CCLE database. **(D)** Relative expression of SLC16A1 in different types of macrophages. **(E)** Kaplan-Meier analysis (log-rank test) showing the OS curves based on the different groups of HCC patients (TCGA database) with different levels of SLC16A1 and infiltration of macrophages.** (F)** Representative images of HCC tissue stained by IHC. Scale bars, 20 μm. CD206^+^ M2 macrophages, CD86^+^ M1 macrophages in human HCC tissue samples (T) (n = 92), and paired normal adjacent tissue samples (N). Two-tailed paired t-test was used to analyze the MFI of SLC16A1 staining in indicated macrophages. **(G)** Relative expression of M2 markers (CD206, ARG1, CD163) in macrophages after treatment with MHCC97H-derived exosomes alone or combined with SLC16A1 knockdown. **(H)** Relative expression of M2 markers (CD206, ARG1, CD163) in macrophages after treatment with MHCC97H-derived exosomes, SLC16A1-A S1 knockdown exosomes alone or combined with SLC16A1 overexpression.

Numerous researches have shown that antisense lncRNAs exert function by regulating its sense gene 27, 28. Since SLC16A1 is a sense coding gene of SLC16A1-AS1 (Fig. 3A), we hypothesized whether the exosomal SLC16A1-AS1 induced M2 polarization of macrophages by regulating SLC16A1 expression in macrophages.

Coincidently, TCGA and CCLE database showed a strong positive correlation between SLC16A1-AS1 and SLC16A1 in HCC tissues (Fig. 3B) and various cancer cell lines (Fig. 3C). Additionally, analysis of the TCGA database indicated that SLC16A1 expression was highest in M2 macrophages compared to M0 and M1 macrophages (Fig. 3D). TIMER2.0 database (http://timer.comp-genomics.org/) displayed that SLC16A1 was positively correlated with M2 markers (CD206 and CD163), but not with macrophage general markers (CD68) and M1 macrophage markers (CD80, CD86, iNOS) in HCC tissues (Supplementary Fig. S3A). Meanwhile, analyzing with the online database TIMER2.0 revealed that SLC16A1 expression was positively associated with the infiltration of M2 macrophages, but not with the infiltration of M1 macrophages (Supplementary Fig. S3B). Furthermore, upon analyzing SLC16A1 expression in THP-1-derived macrophages, M2 polarization showed the highest expression levels (Supplementary Fig. S3C). MoreoverIn addition, we found that HCC patients with high SLC16A1 expression and M2 macrophages infiltration demonstrated the worst prognosis (Fig. 3E). These above results indicated the interaction between SLC16A1 and M2 macrophages in HCC. To compare SLC16A1 expression in M1/M2 macrophages, normal liver tissues and paraffin slides of human liver cancer tissue were stained with fluorescent antibodies to identify the SLC16A1 expression in CD206^+^ M2 macrophages and CD86^+^ M1 macrophages. The mean fluorescence intensity (MFI) of SLC16A1 in CD206^+^ M2 macrophages was stronger than that in control cells of paired normal adjacent tissues. However, the MFI of SLC16A1 in CD86^+^ M1 macrophages did not differ between HCC tissues and normal liver tissues (Fig. 3F). Meanwhile, knockdown or overexpression of SLC16A1-AS1 obviously decreased or increased expression of SLC16A1 in macrophages, respectively (Supplementary Fig. S3D, E). Moreover, incubation with HCC cells-derived exosomes induced remarkably upregulation of SLC16A1 and M2 polarization of macrophages, which was obviously inhibited by SLC16A1 knockdown with siRNA (Fig. 3G and Supplementary Fig. S3F). On the contrary, incubation with exosomes derived from SLC16A1-AS1 knockdown HCC cells could not induce SLC16A1 upregulation and M2 polarization in macrophages, while it was distinctly rescued by overexpression with SLC16A1 (Fig. 3H and Supplementary Fig. S3F). Therefore, these results suggest that SLC16A1 is a critical target for the HCC exosomal SLC16A1-AS1 inducing M2 polarization of macrophage.

### SLC16A1 induces M2 polarization of macrophage via enhancing lactate influx

**Figure 4:**
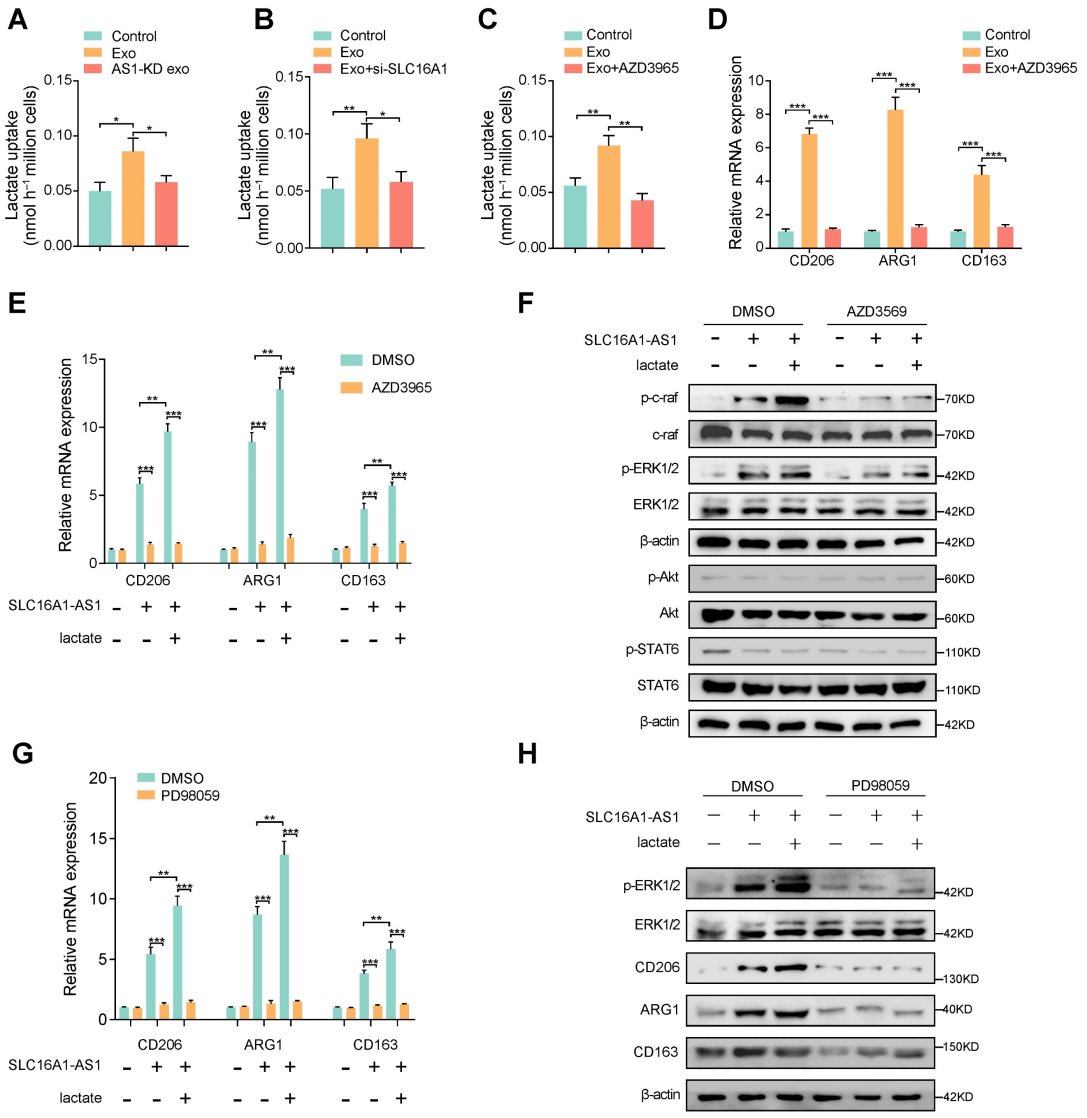
** SLC16A1 induces M2 polarization of macrophage via enhancing lactate influx. (A-C)** Lactate uptake in macrophages after stimulation with MHCC97H-derived exosomes or SLC16A1-AS1 knockdown exosomes or MHCC97H-derived exosomes supplemented with SLC16A1 knockdown, MCT1 inhibitor AZD3965. **(D)** The level of M2 makers (CD206, ARG1, CD163) in macrophages treated with MHCC97H-derived exosomes alone or combined with AZD3965. **(E)** QRT-PCR showing the expression of M2 makers in macrophages transfected with vector or SLC16A1-AS1, and those treated with 20mM lactate or AZD3965. **(F)** Western blot analysis of macrophages transfected with vector or SLC16A1-AS1, and those treated with 20mM lactate in the presence or absence of AZD3965. **(G)** QRT-PCR showing the expression of M2 makers in macrophages transfected with vector or SLC16A1-AS1, and those treated with 20mM lactate or ERK1/2 inhibitor PD98059. **(H)** Western blot analysis of macrophages transfected with vector or SLC16A1-AS1, and those treated with 20mM lactate or ERK1/2 inhibitor PD98059.

SLC16A1 plays an essential role in lactate/pyruvate transport between cells 29, and reports have shown that lactate is essential in regulating M2 macrophage polarization 30, 31. Therefore, we hypothesized that exosomal SLC16A1-AS1 mediates macrophages M2 polarization via SLC16A1-mediated lactate influx. To verify this conjecture, we examined lactate uptake by macrophages under different conditions. Coincidently, lactate uptake in macrophages was increased after incubation with exosomes derived from MHCC97H cells, but not with exosomes derived from MHCC91H cells with SLC16A1 knockdown (Fig. 4A). Furthermore, the HCC exosomes-increased lactate uptake in macrophages could be obviously attenuated by treatment with SLC16A1 knockdown or specific inhibitor of SLC16A1 (AZD3965) (Fig. 4B, C). Moreover, treatment of macrophages with AZD3965 significantly impeded the M2 polarization of macrophages induced by MHCC97H-derived exosome (Fig. 4D). Meanwhile, the addition of 20mM exogenous lactate further reinforced the M2 polarization of macrophages induced by HCC exosomes, which was obviously inhibited by AZD3965 (Fig. 4E). The polarization of macrophages often involves the activation of various signal pathways, which including PI3K/AKT, STAT6, and Raf/ERK 14, 32, 33. Thereafter, we examined whether those three pathways participated in the HCC exosomal SLC16A1-AS1-induced M2 polarization of macrophages. Results showed that the phosphorylation of c-Raf and ERK1/2 obviously increased in macrophages incubated with MHCC97H-derived exosomes, which could be enhanced or inhibited by the addition of exogenous 20 mM lactate or AZD3965. However, neither the phosphorylation of AKT nor the phosphorylation of STAT6 in macrophages was changed under the identical treatment (Fig. 4F). Moreover, the incubation of HCC exosomes or lactate-induced M2 polarization of macrophages was remarkably prohibited by treatment with ERK1/2 inhibitor PD98059 (Fig. 4G, H). In brief, these data indicate that exosomal SLC16A1-AS1 promotes SLC16A1-mediated lactate influx into macrophages and further induces M2 polarization by specifically activating c-raf/ERK signaling.

### Exosomal SLC16A1-AS1 stabilization SLC16A1 mRNA by recruiting HNRNPA1

**Figure 5:**
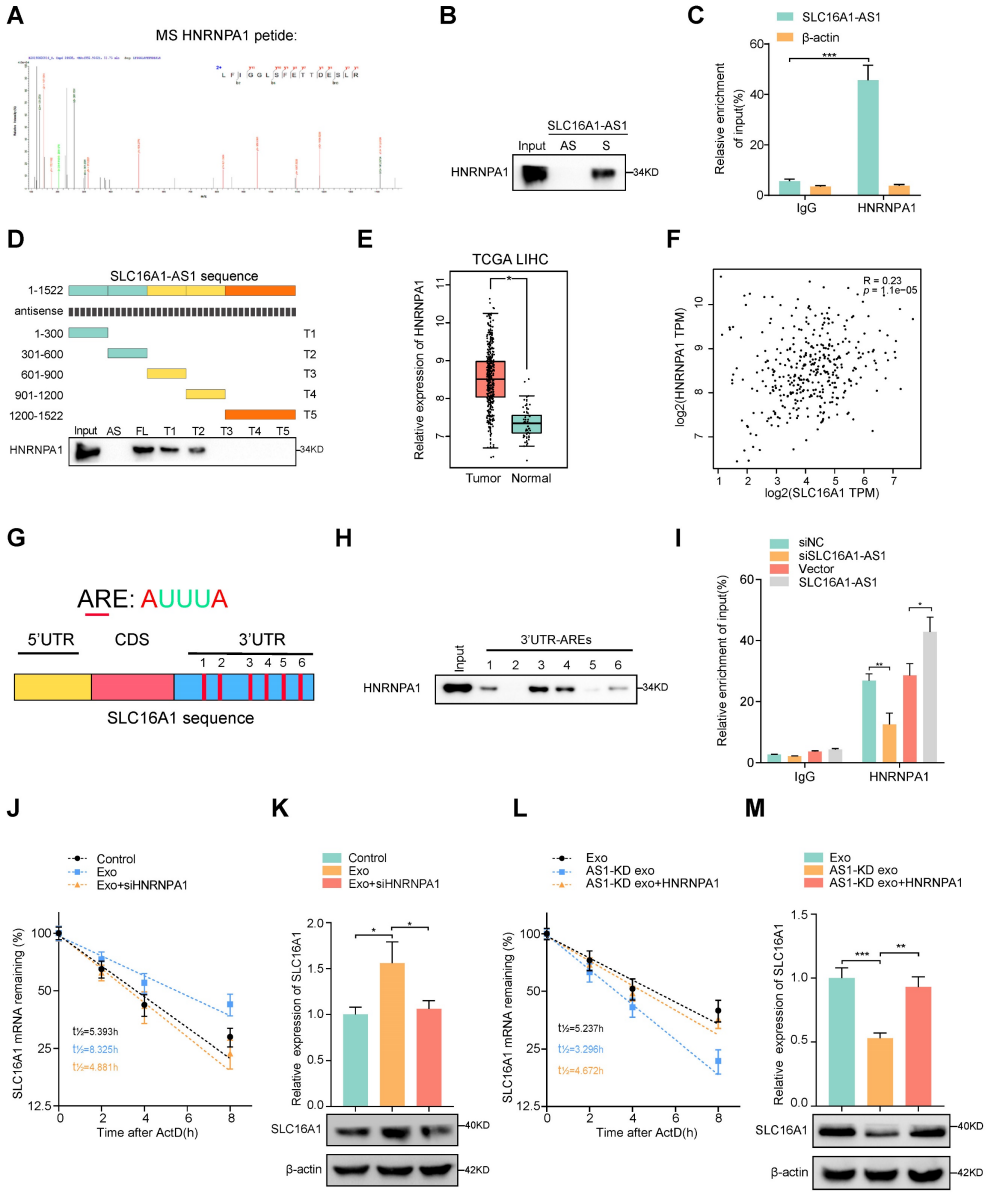
** Exosomal SLC16A1-AS1 stabilization SLC16A1 mRNA by recruiting HNRNPA1. (A, B)** Mass Spectrometry (MS) assay and biotin-labeled RNA pull-down showing the interaction between SLC16A1-AS1 and HNRNPA1 protein in macrophages. The SLC16A1-AS1 antisense (AS) bound protein served as negative control. **(C)** RIP assays were performed using the HNRNNPA1 and IgG antibodies to probe SLC16A1-AS1. β-actin served as a negative control. **(D)** Western blot depicting the recovered HNRNPA1 levels from cell lysates pulled down by biotin-labeled SLC16A1-AS1 truncates. The SLC16A1-AS1 antisense (AS) served as negative control. **(E)** Expression levels of HNRNPA1 were derived from the TCGA-LIHC database. **(F)** Correlation analysis of HNRNPA1 with SLC16A1 in HCC. **(G)** Schematic representation of potential sites of HNRNPA1 binding to SLC16A1 mRNA sequence. Red vertical bars represent AREs (AU-rich elements). **(H)** Western blot depicting the HNRNPA1 levels from cell lysates pulled down by biotin-labeled regions containing AREs of 3'UTR. **(I)** RIP assays were performed using the HNRNNPA1 and IgG antibodies to probe SLC16A1 after SLC16A1-AS1 knockdown or overexpression. **(J, K)** The SLC16A1 mRNA half-life (t_1/2_) or expression levels in macrophages incubated with MHCC97H-derived exosomes alone or combined with HNRNPA1 knockdown. **(L, M)** The SLC16A1 mRNA half-life (t_1/2_) or expression levels in macrophages incubated with AS1-KD exosomes alone or combined with HNRNPA1 overexpression.

To further explore whether SLC16A1-AS1 regulates SLC1A61 expression at the transcriptional level, a luciferase reporter vector containing the SLC16A1 promoter region was transfected into macrophages. Interestingly, neither overexpression nor knockdown of SLC16A1-AS1 in macrophages affected the luciferase activities of the reporter vector (Supplementary Fig. S4A). Therefore, these results indicated that SLC16A1-AS1 regulated SLC16A1 expression in the post-transcriptional level. Since numerous reports have demonstrated that lncRNAs can regulate mRNA stability 34, we further investigated the effects of SLC16A1-AS1 on the stability of SLC16A1 mRNA. Following treatment with RNA polymerase activity inhibitor ActD which inhibited the synthesis of mRNA, results showed that the half-life of SLC16A1 mRNA was markedly shortened after knockdown of SLC16A1-AS1 (Supplementary Fig. S4B), conversely prolonged after overexpression of SLC16A1-AS1 (Supplementary Fig. S4C). We subsequently performed RNA pulldown assay followed by mass spectrometry using in vitro transcribed biotinylated SLC16A1-AS1 and an antisense control to identify SLC16A1-AS1 interacting proteins in macrophages. Results of mass spectrometry demonstrated that sense SLC16A1-AS1, but not the antisense control, interacted specifically with heterogeneous nuclear ribonucleoprotein A1 (HNRNPA1) (Fig. 5A and Table S5), which was further validated by RNA pulldown assay (Fig. 5B). Meanwhile, the interaction between SLC16A1-AS1 and HNRNPA1 was also confirmed by RNA immunoprecipitation (RIP) assays (Fig. 5C). Moreover, the deletion-mapping analyses suggested that HNRNPA1-binding site was located in the first 600nt of SLC16A1-AS1 (Fig. 5D). Moreover, analysis from TCGA revealed that HNRNPA1 is obviously overexpressed in LIHC (Fig. 5E), and other gastrointestinal tumors including COAD, CHOL, ESCA, pancreatic adenocarcinoma (PAAD), and rectum adenocarcinoma (READ) (Supplementary Fig. S4D). Furthermore, high expression of HNRNPA1 was correlated with the high grade and stage of HCC samples, as well as DFS and OS in HCC patients (Supplementary Fig. S4E, F). Nevertheless, HNRNPA1 expression is positively associated with SLC16A1 in HCC and HCC cell lines (Fig. 5F and Supplementary Fig. S4G). Nevertheless, neither knockdown nor overexpression of SLC16A1-AS1 affected the expression of HNRNPA1 (Supplementary Fig. S4J, K). Coincidently, knockdown of HNRNPA1 significantly downregulated, while overexpression of HNRNPA1 obviously upregulated the mRNA and protein levels of SLC16A1 (Supplementary Fig. S4L, M). These data intensively indicated that SLC16A1-AS1 regulates the stability of SLC16A1 mRNA via binding to HNRNPA1.

HNRNPA1 had been recovered to promote the stability of mRNA by binding to adenylate/uridylate-rich elements (AREs) in the 3' untranslated region (3'UTR) 35, 36. Meanwhile, bioinformatics analysis revealed 6 AREs in the 3'UTR of SLC16A1 mRNA, indicating a potential interaction between SLC16A1 mRNA and HNRNPA1 (Fig. 5G). Coincidently, the RNA pulldown assay revealed that ARE-3, 4 in 3'UTR were the primary binding sites for HNRNPA1 (Fig. 5H). Moreover, RIP assays also proved that SLC16A1 mRNA was enriched by antibody against HNRNPA1, which was increased or decreased individually by overexpression or knockdown of SLC16A1-AS1 (Fig. 5I). After treatment with ActD in macrophages, the prolonged half-life of SLC16A1 mRNA by HCC cell-derived exosomes was remarkably shortened by knockdown of HNRNPA1(Fig. 5J, K and Supplementary Fig. S4H). In contrast, overexpression of HNRNPA1 increased the mRNA stability of SLC16A1 in macrophages incubated with AS1-KD exosomes (Fig. 5l, M and Supplementary Fig. S4I) Taken together, these results intensively indicated that HCC exosomal SLC16A1-AS1 promotes HNRNPA1-mediated stability of SLC16A1 mRNA.

### M2 macrophages increases SLC16A1-AS1 expression in HCC cells by inducing METTL3-mediated m^6^A modification

**Figure 6:**
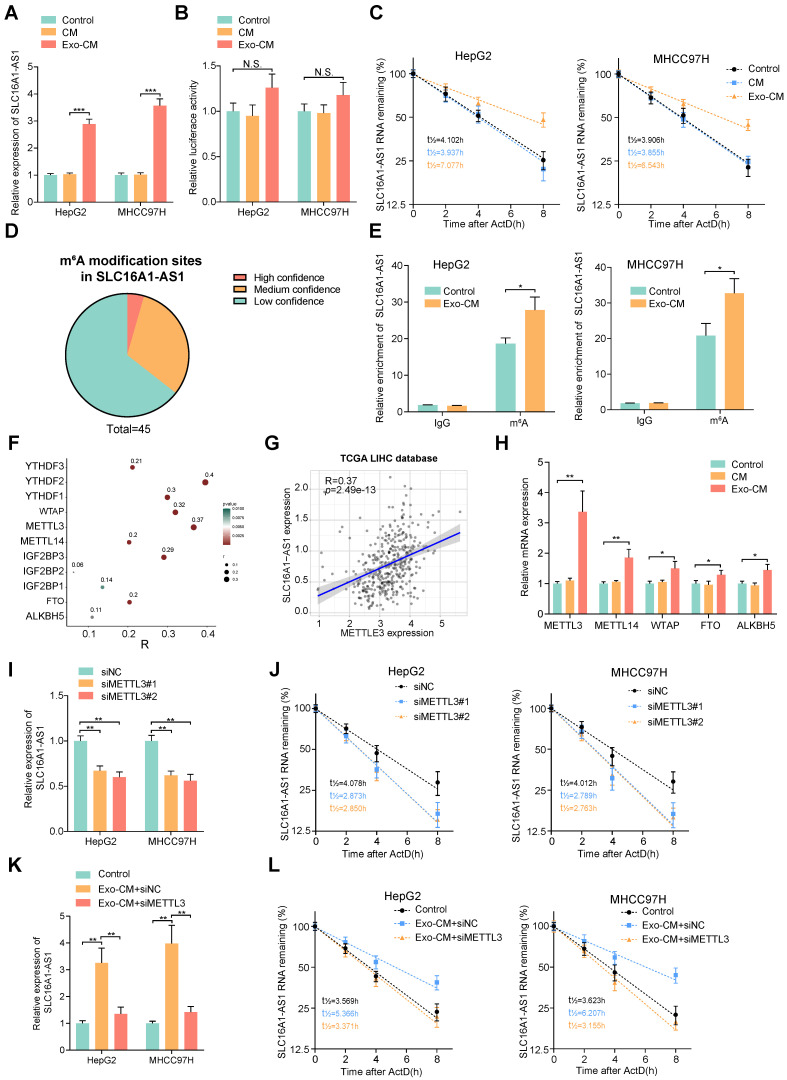
** M2 macrophages increases stabilization of SLC16A1-AS1 by regulating METTL3-mediated m^6^A modification. (A)** Relative expression of SLC16A1-AS1 in HCC cells after incubation with CM or Exo-CM. **(B)** The luciferase activity in the promoter region of SLC16A1-AS1 in HCC cells after incubation with CM or Exo-CM. **(C)** The SLC16A1-AS1 half-life (t_1/2_) was detected by qRT-PCR in HCC cells incubation with CM or Exo-CM. **(D)** Pie chart of m^6^A modification sites of SLC16A1-AS1. **(E)** m^6^A RIP-qPCR analysis showed that m^6^A enrichment within SLC16A1-AS1 in HCC cells incubated with Exo-CM or not. **(F)** Scatter plots showing the association of SLC16A1-AS1 with m^6^A-related enzymes. **(G)** Correlation analysis of SLC16A1-AS1 and METTL3 in HCC. **(H)** Relative expression of m^6^A reader or eraser in MHCC97H cells after incubation with CM or Exo-CM. **(I-L)** Relative expression and half-life (t_1/2_) of SLC161A-AS1 after knockdown of METTL3 in HCC cells in the presence or absence of Exo-CM.

Since numerous researches have suggested a crosstalk between tumor cells and macrophages, we further investigated whether the M2 polarization of macrophages could reciprocally regulate the SLC16A1-AS1 expression of HCC cells. Coincidently, we observed that the expression of SLC16A1-AS1 was elevated in MHCC97H and HepG2 cells co-cultured with conditional medium (CM) from exosome-exposed macrophages (Exo-CM) compared to CM from M0 macrophages (Fig. 6A). After transfection with luciferase reporter vector containing a promoter sequence of SLC16A1-AS1, the transcription activity of SLC16A1-AS1 in HCC cells was evaluated. Interestingly, the luciferase activity of HCC cells did not change obviously after incubated with Exo-CM (Fig. 6B). Nevertheless, after treated with ActD, stability of SLC16A1-AS1 of HCC cells was significantly increased in HepG2 and MHCC97H cells which were incubated with Exo-CM (Fig. 6C). These results suggest that CM of M2 polarized macrophages regulates HCC SLC16A1-AS1 expression in post-transcriptional level.

N^6^-methyladenosine (m^6^A) is the most abundant internal RNA modification in eukaryotic systems 37. The latest advances in the epigenetic regulation of tumors have revealed that the m^6^A modification affects lncRNA stabilization 38. Thereafter, we wondered whether m^6^A modification was involved in the Exo-CM-induced upregulation of SLC16A1-AS1 in HCC cells. Through the online database m6var 39, we found numerous potential m^6^A modification sites in SLC16A1-AS1 (Fig. 6D). Meanwhile, MeRIP-qPCR assays identified that SLC16A1-AS1 in HCC cells were distinctively enriched by m^6^A antibody but not the IgG antibody, which was enhanced after incubation with Exo-CM (Fig. 6E). N^6^-methyladenosine is a dynamic process controlled by the catalytic activities of the m^6^A writer and eraser enzymes, and the m^6^A readers directly determine the outcome of m^6^A methylated RNA 40. The expression pattern of m^6^A-related enzymes was analyzed in LIHC based on TCGA datasets showed that m^6^A-related enzymes were all upregulated in LIHC (Supplementary Fig. S5A, B).

Among methyltransferases and demethylases, methyltransferase-like 3 (METTL3) had the most significant correlation with SLC16A1-AS1 in LIHC (Fig. 6F, G). Furthermore, results of qRT-PCR further verified that METTL3 significantly increased methyltransferases in MHCC97H cells incubated with Exo-CM (Fig. 6H). Moreover, survival analysis from TCGA demonstrated that HCC patients with high METTL3 expression had both shorter DFS and OS (Supplementary Fig. S5C). Moreover, knockdown of METTL3 obviously reduced the expression and stability of SLC16A1-AS1(Fig. 6I, J). In addition, the increased expression and stability of SLC16A1-AS1 in HCC cells induced by Exo-CM were apparently reversed by knockdown of METTL3 (Fig. 6K, L).

### IGF2BP3 is required for the M2 macrophages-mediated m^6^A modification and stability of SLC16A1-AS1 in HCC cells

Recently, studies have reported that insulin-like growth factor binding protein families (IGF2BPs; IGF2BP1/2/3) function as m^6^A readers and are related to the regulation of RNA stability^41^. Intriguingly, analysis of* the cat*RAPID database suggested an intensive binding potential of IGF2BPs to SLC16A1-AS1 (Supplementary Fig. S5D). Among the IGF2BPs, IGF2BP3 showed the highest correlation with SLC16A1-AS1 in the HCC dataset from TCGA (Fig. 6F and Supplementary Fig. S5E). Meanwhile, RIP analysis showed that SLC16A1-AS1 was enriched on the anti-IGF2BP3 antibody, which was significantly inhibited after knockdown of METTL3 in HCC cells (Supplementary Fig. S5F). Furthermore, we noticed that knockdown of IGF2BP3 observably decreased the expression and stability of SLC16A1-AS1 in HepG2 and MHCC97H cells (Supplementary Fig. S5G, H). Coincidently, the Exo-CM-induced expression and stabilization of SLC16A1-AS1 in HCC cells was evidently decreased by knockdown of IGF2BP3 (Supplementary Fig. S5I, J). Collectively, these results remind us that M2 macrophages increase SLC16A1-AS1 expression of HCC cells through METTL3-mediated m^6^A modification and IGF2BP3-dependent RNA stability.

### M2 macrophages induces METTL3 expression in HCC cells through IL-6/STAT3 signaling pathway

**Figure 7:**
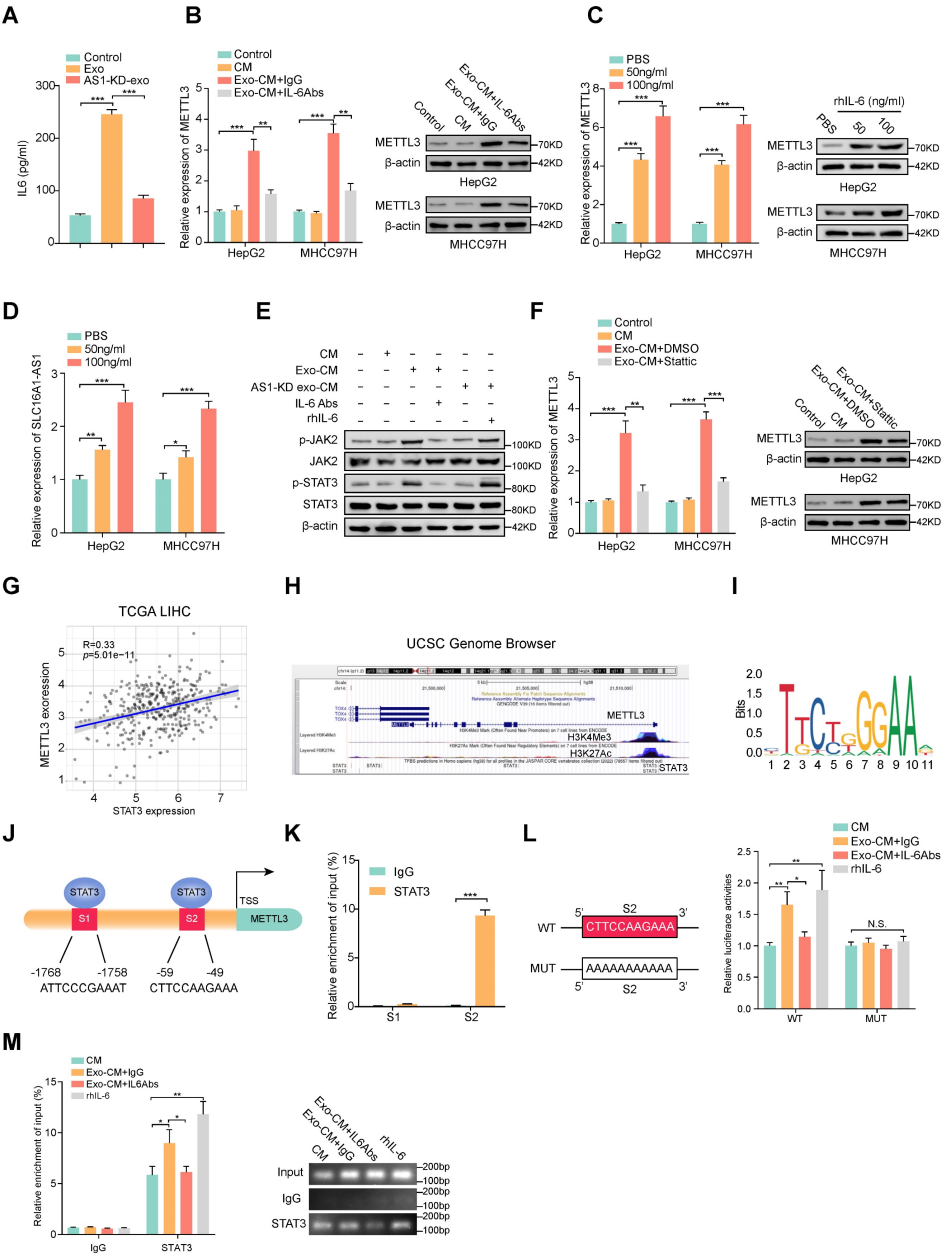
** M2 macrophages regulate the expression of METTL3 through IL-6/STAT3 signaling pathway. (A)** ELISA was used to measure the level of IL-6 protein in macrophages incubated with MHCC97H-derived exosomes or SLC16A1AS-1 knockdown exosomes. **(B)** The mRNA (left) and protein (right) levels of METTL3 in HCC cells incubated with CM, Exo-CM with IgG, or Exo-CM with IL-6Ab, respectively. **(C)** The mRNA (left) and protein (right) levels of METTL3 in HCC cells were treated with different concentrations of IL-6 cytokine. **(D)** The levels of SLC16A1-AS1 in HCC cells treated with different concentrations of IL-6 cytokine. **(E)** Western blot analysis of MHCC97H cells alone, MHCC97H cells incubated with CM, Exo-CM, Exo-CM with IL-6Abs or AS1-KD exo-CM with rhIL-6. **(F)** The mRNA (left) and protein (right) levels of METTL3 in HCC cells alone, HCC cells incubated with CM, Exo-CM or Exo-CM with Stattic. **(G)** Correlation analysis of METTL3 and STAT3 in HCC. **(H)** ChIP-Seq tracks from the UCSC database (http://genome.ucsc.edu/) show the enrichments of H3K4me3, H3K27ac, and STAT3 across the METTL3 promoter sequence. **(I)** Prediction of the consensus STAT3 binding sequence was presented via the JASPAR database. **(J)** The illustration shows the binding site of STAT3 to the METTL3 promoter region. **(K)** Relative enrichment of STAT3 to METTL3 promoter was detected by ChIP assays in MHCC97H cells. **(L)** Schematic representation of the mutant sequence (left). Luciferase reporter assays show the relative activity of METTL3 promoter in MHCC97H cells containing WT or MUT reporter vector. These cells were further treated with CM, Exo-CM, and Exo-CM with IL-6Abs, or rhIL-6 (right). **(M)** Relative enrichment of STAT3 to METTL3 promoter was detected by ChIP assays in MHCC97H cells treated with CM, Exo-CM, Exo-CM+IL-6Abs, or rhIL-6 (left). The PCR products were run on a 2% agarose gel (right).

ELISA results displayed that HCC exosomes induced the most significant elevation of IL-6 concentration in CM of macrophages (Fig. 7A and Supplementary Fig. S6A), we further investigated whether the increase of METTL3 in HCC cells was induced by M2 macrophages-derived IL-6. Coincidently, the Exo-CM-induced METT3 upregulation in HCC cells was evidently attenuated by the addition of exogenous IL-6 neutralizing antibodies (IL-6 Abs) (Fig. 7B). To further evaluate whether IL-6 was critical for the upregulation of METTL3 in HCC cells, an exogenous recombinant human IL-6 (rhIL-6) was added in the culture medium of HCC cell lines. The results showed that rhIL-6 significantly increased the expression of METTL3 and SLC16A1-AS1 of HCC cells in a dose-dependent manner (Fig. 7C, D). It is well accepted that JAK2/STAT3 is a critical target of IL-6, therefore, we further explored whether IL-6 promoted METTL3 expression by activating JAK2/STAT3 pathway. Coincidently, the phosphorylation of JAK2 and STAT3 was significantly increased in HCC cells incubated with Exo-CM, which was further suppressed by treatment with IL-6 Abs (Fig. 7E). Furthermore, treatment with STAT3 inhibitor Stattic reverses the upregulation of METTL3 expression in HCC cells which was caused by incubation with Exo-CM (Fig. 7F). Meanwhile, the analysis from TCGA showed that METTL3 expression was positively correlated with STAT3 in HCC (Fig. 7G). Notably, the UCSC Genome Browser database revealed a significant enrichment of STAT3 at the METTL3 promoter region accompanied by enriched histone modification peaks (H3K4Me3 and H3K27Ac) which are indicative of an active transcription (Fig. 7H). The JASPAR database also revealed 2 putative sites for STAT3 binding motifs in the promoter region of METTL3 (Figure 7I, J). Meanwhile, the ChIP assay showed obvious enrichment of binding site 2 (S2) by anti-STAT3 antibody but not S1 (Fig. 7K). Meanwhile, luciferase reporter vectors containing METTL3 promoter sequences containing wild-type (WT) or mutant S2 (MUT) were transfected into MHCC97H cells individually to evaluate the transcription activity of STAT3 on METTL3. The results showed that rhIL-6 or Exo-CM could enhance the luciferase activity of the METTL3 promoter of the WT cells, whereas no noticeable change was observed in the MUT cells (Fig. 7L). Furthermore, supplemented with exogenous IL-6 Abs weakened the luciferase activity of METTL3 promoter caused by Exo-CM (Fig. 7L). Subsequently, ChIP assay results also verified that the enrichment of STAT3 on METTL3 promoter was enhanced by the addition of rhIL-6 or Exo-CM and further inhibited by combination with exogenous IL-6 Abs (Fig. 7M). Together, these data remind us that M2 macrophage-derived IL-6 promotes the transcription of METTL3 by activating the JAK2/STAT3 signaling pathway which continually increases SLC16A-AS1 expression in HCC cells.

### HCC exosomes-incubated macrophages enhance proliferation, metastasis, and glycolysis of HCC

Continually, we further evaluate the effects of HCC exosomes-induced M2 macrophages on the proliferation and metastasis of HCC cells. Knockdown of SLC16A1-AS1 alone in HCC cells did not affect their proliferation and invasion ability (Supplementary Fig. S7A, B). However, colony formation and transwell assays revealed that the incubation with Exo-CM apparently promoted the proliferative and invasive ability of HCC cells, which were attenuated by the exogenous addition of IL-6 Abs (Supplementary Fig. S7C, D). Moreover, SLC16A1-AS1 knockdown exosomes-exposed macrophages CM (AS1-KD Exo-CM) had no significant effect on the proliferation and invasion of HCC cells, while the addition of exogenous rhIL-6 significantly increased the above effects (Supplementary Fig. S7C, D). Metabolic reprogramming based on aerobic glycolysis is a characteristic of HCC 42. Moreover, STAT3 signaling has been reported to regulate glycolysis in HCC 43. Therefore, we next explored the effect of IL-6/STAT3 on glycolysis in HCC. Interestingly, the level of glucose uptake and lactate production of HCC cells were significantly increased after incubation with Exo-CM, which was inhibited by IL-6 neutralizing antibody treatment (Supplementary Fig. S7E, F). Similarly, glucose uptake and lactate production were significantly increased in rhIL-6-treated HCC cells, while these effects were attenuated by STAT3 pathway inhibition Stattic (Supplementary Fig. S7G, H). These results suggested that IL-6 cytokines secreted by M2 macrophages promoted glycolysis of HCC by activating the STAT3 signaling pathway.

**Figure 8:**
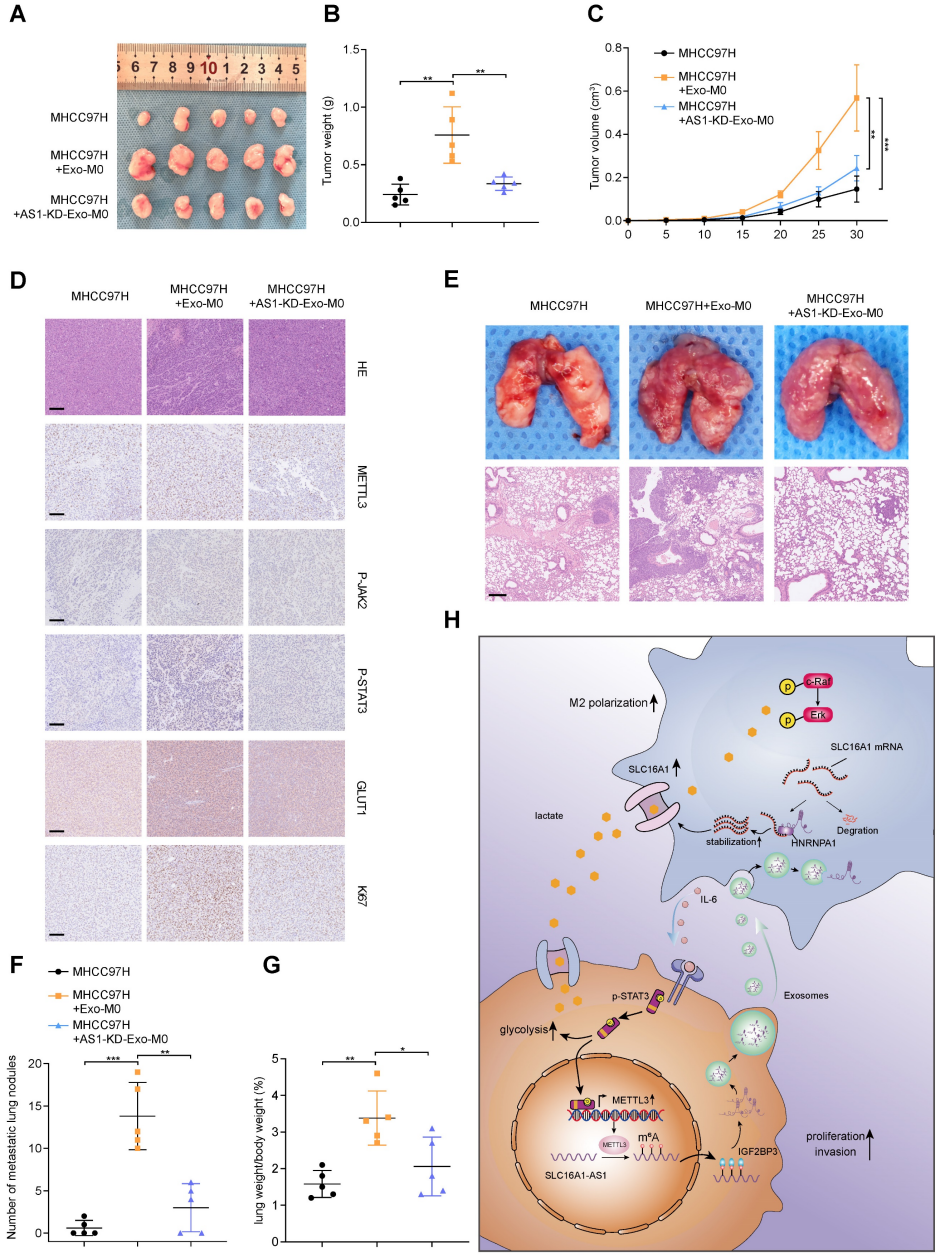
** HCC exosomes-incubated macrophages enhance proliferation, metastasis, and glycolysis. (A-C)** The morphological characteristics of tumor xenograft, tumor weight, and tumor volume in the indicated groups. **(D)** IHC analyzed the expression of METTL3, p-JAK2, p-STAT3, GLUT1, and Ki67 protein of tumors from the indicated groups. Scale bar: 100 μm. **(E)** Representative images of lung metastasis in nude mice that resulted from the indicated groups. Scale bar: 200 μm. **(F)** The number of metastatic nodules in each indicated group. **(G)** The lung weight/body weight (%) in each indicated group. **(H)** Schematic illustration of HCC cell-derived exosomes promoted the M2 polarization of macrophages and malignant progression of HCC.

To verify the in vitro findings, an in vivo assay was performed. MHCC97H cells alone, Exo-incubated M0 macrophages and MHCC97H cells, AS1-KD Exo-incubated M0 macrophages and MHCC97H cells or MHCC97H cells alone, M2 macrophages/ siNC and MHCC97H cells, M2 macrophages/siIL-6 and MHCC97H cells were separately injected into the flanks of female nude mice to conduct xenograft model (Fig. 8A and Supplementary Fig. S8A). Compared with MHCC97H cells alone, MHCC97H cells mixed with Exo-incubated macrophages or M2 macrophages displayed heavier tumor weight and larger tumor volume. However, both tumor weight and volume were reduced in the Exo-incubated M0 macrophages and MHCC97H cells or M2 macrophages/siIL-6 and MHCC97H cells groups. (Fig. 8B, C and Supplementary Fig. S8B, C). Moreover, the IHC staining also confirmed that METTL3, p-JAK2/p-STAT3, GLUT1, and Ki67 were significantly elevated in the mice tumor with injection of MHCC97H mixed with Exo-incubated macrophages or M2 macrophages. (Fig. 8D and Supplementary Fig. S8D).

To confirm the effect of macrophages on tumor metastasis in vivo, the same groups were carried out as described above and were injected into nude mice via the tail vein. After 4 weeks, we observed that MHCC97H mixed with Exo-incubated macrophages or M2 macrophages group generated more and larger nodules of metastatic tumors in the lung than the other two groups. (Fig. 8E-G). These results suggest that HCC exosomal SLC16A1-AS1-induced M2 polarization of macrophages contributes to the proliferation, metastasis, and glycolysis of HCC.

Our study illustrated a crosstalk between M2 macrophages and cancer cells in the HCC microenvironment, and the findings were summarized with a schematic (Fig. 8H). HCC-cell-derived exosomal SLC1A61-AS1 upregulated the lactate transporter SLC16A1 of macrophages and induced the intracellular influx of lactate to activate the ERK signaling pathway and promoted the activation of M2 macrophages, which in turn activated M2 macrophages secrete IL-6 and other cytokines act on adjacent HCC cells to promoted its proliferation and metastasis. Moreover, by activating the JAK-STAT3 signaling pathway, IL-6 promoted the glycolysis level and increased the expression of SLC16A1-AS1 in HCC cells, thereby forming a positive feedback loop.

### SLC16A1-AS1 and SLC16A1 are highly expressed in HCC and correlated with M2 polarization of macrophages

**Figure 9:**
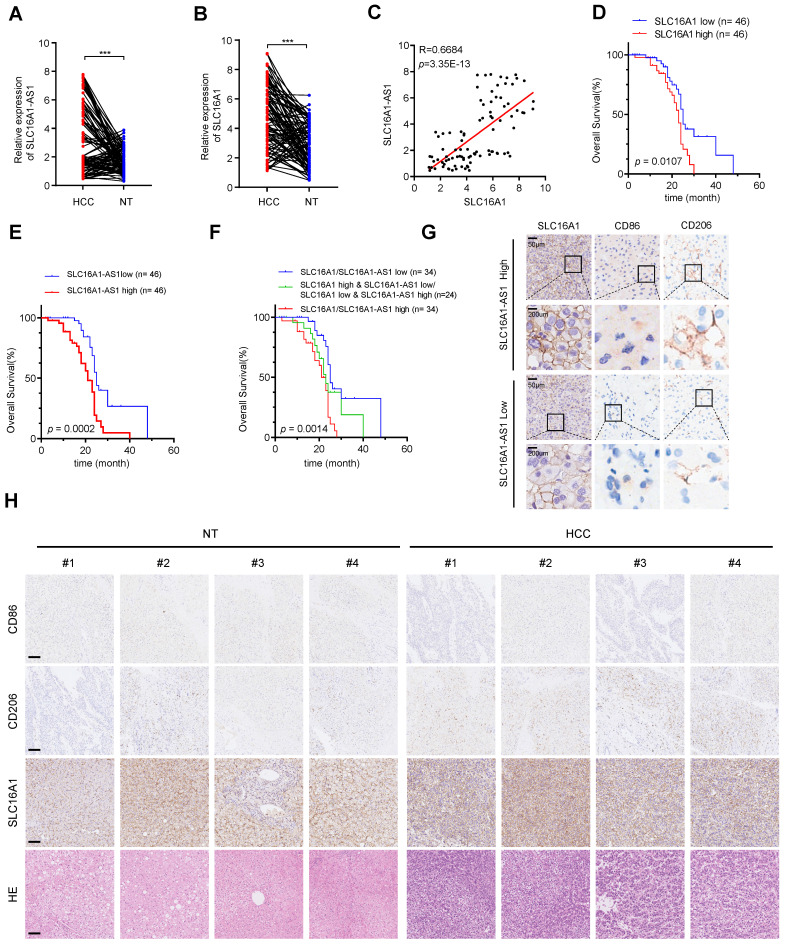
** SLC16A1-AS1 and SLC16A1 are highly expressed in HCC and demonstrate a strong positive correlation with M2 macrophages. (A-B)** The expression of SLC16A1-AS1 and SLC16A1 in 92 pairs of HCC tissues and paired paracancerous tissues. **(C)** Correlation between SLC16A1-AS1 and MCT1 in 92 HCC tissues. **(D-E)** Kaplan-Meier analyses of overall survival (OS) in 92 HCC tissues with low and high levels of MCT1 or SLC16A1-AS1 expression using the log-rank test. **(F)** Kaplan-Meier analysis was used to analyze the overall survival (OS) of 92 HCC tissues with combined survival of SLC16A1/SLC16A1-AS1. **(G)** The representative IHC images showed SLC16A1/CD206/CD86 expression in HCC tissues with high or low SLC16A1-AS1. **(H)** Representative IHC staining of CD86, CD206, and SLC16A1 in HCC and paracancerous tissues, respectively. Scale bar: 100 μm.

To further confirm the previous findings, we then examined the expression of SLC16A1-AS1 and SLC16A1 in 92 paired HCC and paracancerous tissues. SLC16A1-AS1 and SLC16A1 were highly expressed in HCC tissues compared with paired adjacent tissues (Fig. 9A, B). Additionally, there was a significant positive correlation observed between SLC16A1-AS1 and SLC16A1 (Fig. 9C).

Furthermore, HCC patients with higher SLC16A1-AS1 or SLC16A1 expression displayed a poorer OS (Fig. 9D, E). Further survival analysis showed that a low level of both SLC16A1-AS1 and SLC16A1 was much more beneficial to OS of HCC patients, followed by a low level of either SLC16A1-AS1 or SLC16A1, while a high level of both SLC16A1-AS1 and SLC16A1 was unfavorable for prognosis (Fig. 9F). The immunohistochemical (IHC) staining showed that the high expression of SLC16A1-AS1 was associated with elevated levels of SLC16A1 and CD206 while exhibiting reduced expression of CD86 in HCC tissues (Fig. 9G and Supplementary Fig. S9A). In addition, high expression of SLC16A1 was associated with high expression of CD206 and low expression of CD86 (Supplementary Fig. S9A). The IHC staining suggested that the expression levels of SLC16A1 and CD206 were found to be significantly elevated in HCC tissues compared to non-tumor tissues, whereas the expression level of CD86 was relatively diminished in HCC tissues (Fig. 9H). Moreover, SLC16A1-AS1 or SLC16A1 expression was positively correlated with CD206 in HCC patients (Supplementary Fig. S9B). Collectively, our findings present novel insights for the diagnosis and therapeutic strategies of HCC, with a potential focus on targeting SLC16A1-AS1/SLC16A1.

## Discussion

In this study, by analyzing the TCGA database, we found that M2 macrophages were the most abundant infiltrating cells in HCC and were associated with poor prognosis. Therefore, exploring potential mechanisms regulating macrophages M2 polarization in HCC may provide new perspectives for the treatment of HCC. Exosomes are cell-derived vesicles that play an important role in intercellular communication, exchanging cellular materials and information. In this study, we found that lncRNA SLC16A1-AS1 in exosomes secreted by HCC cells can reshape the tumor microenvironment. Exosomal SLC16A1-AS1 could be endocytosed by macrophages and promote the M2 polarization by enhancing SLC16A1-mediated lactate influx into macrophages. Meanwhile, M2 macrophages-secreted IL-6 further promoted m^6^A methylation and stabilization of SLC16A1-AS1 in HCC cells to form a feedforward regulatory loop. Meanwhile, IL-6 enhanced glycolysis of HCC cells by activating STAT3 signaling which further facilitated M2 polarization of macrophages and microenvironment reshape and tumorigenesis of HCC. These findings provide a novel perspective into the interaction between HCC cells and tumor microenvironment.

Research has shown that accumulated lncRNAs can be selectively packaged into exosomes and act as messengers in intercellular communication to reshape the TME and promote tumor progression 44. In our study, to investigate whether HCC cells-derived exosomal lncRNA could regulate macrophage polarization, lncRNA sequencing was performed by extracting exosomes from the supernatant of the HCC cell culture medium. We identified that SLCA16A1-AS1 was highly expressed in exosomes derived from HCC cells. Moreover, M2 polarization of macrophages was induced by overexpression of SLCA16A1-AS1 or incubation with HCC cells-derived exosomes, but not with exosomes derived from SLCA16A1-AS1 knockdown HCC cells. Therefore, our results provide further evidence that exosomal lncRNA contributes to M2 polarization in HCC.

Reports have suggested that natural antisense lncRNA transcripts are widely present in mammalian genomes and play vital roles in the regulation of sense gene expression 45. Since SLC16A1-AS1 is a natural antisense lncRNA for SLC16A1, we wonder whether SLC16A1-AS1 exerts function by targeting SLC16A1 expression. Coincidently, analysis from the TCGA dataset revealed that SLC16A1 was positively correlated with SLC16A1-AS1 expression, and also positively associated with the infiltration abundance of M2 macrophages. Subsequently, we experimentally verified that SLC16A1-AS1 in exosomes promoted M2 polarization by regulating the expression of SLC16A1 in macrophages at the post-transcriptional level.

To further investigate the mechanism by which SLC16A1-AS1 regulates SLC16A1, we performed an RNA pulldown assay and demonstrated that HNRNPA1 is a SLC16A1-AS1 binding protein. HNRNPA1 is a member of a family of ubiquitously expressed heterogeneous nuclear ribonucleoproteins (hnRNPs), which play a pivotal role in cellular RNA processing such as splicing, stability, nuclear export, and translation 46. Pan-cancer analysis revealed that HNRNPA1 was highly expressed in a variety of gastrointestinal tumors including HCC and associated with shorter DFS and OS in HCC patients. HNRNPA1 regulates the stabilization of mRNA by recognizing and binding to AU-rich elements (AREs). Specifically, sequence analysis revealed multiple AREs on SLC16A1 mRNA. Meanwhile, pulldown and RIP assay showed that HNRNPA1 could directly bind to ARE-3,4 in 3'UTR of SLC16A1 mRNA. Knockdown or overexpression of HNRNPA1 correspondingly impaired or improved SLC16A1 mRNA stability. Moreover, overexpression of SLC16A1-AS1 or incubation with HCC exosomes promoted interaction between HNRNPA1 and SLC16A1 mRNA. Nevertheless, knockdown of HNRNPA1 obviously prohibited SLC16A1 expression in macrophages which was induced by overexpression of SLC16A1-AS1 or incubation with HCC exosomes. Therein, our results imply that SLC16A1-AS1 regulates SLC16A1 expression HNRNPA1-mediated mRNA stabilization.

SLC16A1 encodes for monocarboxylate transporter 1 (MCT1) and mediates lactate across cell membranes. Lactate functions as the end product of glycolysis secreted by metabolism-reprogrammed cancer cells and facilitates the establishment of an immunosuppressive TME 47. Studies have shown that lactate can promote the M2 polarization of macrophages. Colegio OR et al. have demonstrated that lactate induced the M2 polarization of macrophages by hypoxia-inducible factor 1α (HIF1α) dependent pathway 30. Research from Zhang J et al. suggested that lactate influx into macrophages promotes M2 polarization in an MCT1-dependent manner and provides energy for oxidative phosphorylation of M2 macrophages 31. We therefore hypothesized exosomal SLC16A1-AS1 might mediate macrophages M2 polarization via SLC16A1-mediated lactate influx. Subsequently, we found that the lactate influx in macrophages was enhanced after being treated with SLC16A1-AS1 overexpression or HCC exosomes. Moreover, lactate combined with SCL16A1-AS1 overexpression further promotes M2 polarization of macrophages, which was inhibited by AZD3965, the SLC16A1-specific inhibitor. These results intensively suggest that SLC16A1-AS1 induces M2 polarization of macrophage by promoting lactate influx. Macrophage polarization is closely related to the activation of signaling pathways 8. In this study, three signaling pathways involved in macrophage polarization including PI3K/AKT, STAT6, and Raf/ERK were analyzed. Specifically, we found that only the Raf/ERK pathway was activated by SLC16A1-AS1 overexpression, which was further activated by combination with lactate.

It is interesting to explore whether exosome-induced M2 macrophages reciprocally promoted the expression of SLC16A1-AS1 in HCC cells. Coincidently, we revealed that SLC16A1-AS1 stabilization and expression were elevated in HCC cells co-cultured with CM from M2 macrophages. N6-methyladenosine (m^6^A) modification in RNA is an essential epigenetic regulation mode in eukaryotes. Some studies suggested that m^6^A modification was important for lncRNA stabilization 38. Since bioinformatics analysis showed m^6^A modification sites in SLC16A1-AS1, we speculated that M2 macrophages enhanced the stability of SLC16A1-AS1 via regulating m^6^A methylation. Meanwhile, the MeRIP experiment verified that SLC16A1-AS1 could be modified by m^6^A methylation, which was further enhanced by co-culture with CM from M2 macrophages. Moreover, we further recovered that M2 macrophage significantly increased the expression of METTL3, while knockdown of METTL3 significantly attenuated the elevation of SLC16A1-AS1 stability in HCC cells induced by M2 macrophages.

Various research demonstrated that polarized M2 macrophages secrete a bunch of cytokines, and chemokines to promote tumor progression 48, thereby, we hypothesized whether the METTL3-mediated m^6^A methylation in HCC cells was induced by cytokine secreted from M2 macrophages. After that, we analyzed the expression of cytokines secreted by exosome-incubated macrophages and found that IL-6 was the most significantly increased cytokine. IL-6 plays an essential regulatory role among the cytokines secreted by macrophages in promoting tumor progression. Wei C et al. have demonstrated that TAM-derived IL-6 activated the JAK2/STAT3 pathway promoting the invasion and metastasis of CRC 49. The report showed TAM-secreted IL-6-induced chemoresistance by activating the IL-6R/STAT3/miR-204 pathway in colorectal cancer cells 50. Coincidingly, we demonstrated that IL-6 neutralizing antibody significantly inhibited the METTL3 expression in HCC cells, which was induced by incubation with CM of M2 macrophages or co-culture with M2 macrophages. Specifically, bioinformatics analysis revealed STAT3 binding sites in the promoter region of METTL3, which was further confirmed by ChIP and luciferase reporter assay. Subsequently, we verified that METTL3 was regulated by IL-6 at the transcriptional level through activation of STAT3. Thus, we uncover that IL-6 functions as a mediator that facilitates the crosstalk between macrophages and HCC cells.

Aerobic glycolysis is a metabolic hallmark of malignancy, some studies have reported that TAMs can promote glycolysis of tumor cells to maintain malignancy 51. Moreover, STAT3 is a critical signaling pathway in the development of HCC 52, and previous studies have confirmed that the STAT3 signaling pathway plays an important role in the regulation of HCC glycolysis 53, 54. Therefore, we wondered whether IL6 secreted by M2 macrophages could further enhance glycolysis in HCC cells. Interestingly, CM from M2 macrophages significantly promoted glucose uptake and lactate production in HCC cells, whereas the addition of IL-6 neutralizing antibodies compromised these effects. Similarly, inhibition of the STAT3 pathway also impaired the enhanced glycolysis induced by IL-6. Moreover, lactate as a product of glycolysis in turn further promoted the M2 polarization of macrophages. These results indicate that IL-6/STAT3 is an important pathway of M2 macrophages in promoting the progression of HCC. Subsequently, we verified that HCC cells-derived exosomal SLC16A1-AS1 accelerated tumor growth and metastasis by promoting M2 polarization of macrophages in animal experiments. Finally, our viewpoint was further substantiated through the analysis of 92 HCC specimens. Remarkably elevated expression levels of SLC1A61 and SLC16A1-AS1 were observed in HCC, correlating with an unfavorable prognosis. Additionally, a positive correlation was found between high SLC1A61 and SLC16A1-AS1 expression and increased CD206 expression in HCC tissues.

## Conclusions

In conclusion, our study of TCGA data revealed that M2 macrophages were predominant in HCC and were associated with poor prognosis. Exosomal lncRNA SLC16A1-AS1 from HCC cells induced M2 polarization by enhancing SLC16A1-mediated lactate influx into macrophages. This formed a feedforward loop via IL-6/STAT3 signaling. The stability of SLC16A1-AS1 was regulated by HNRNPA1-mediated mRNA stabilization. M2 macrophages reciprocally enhanced SLC16A1-AS1 expression in HCC cells, promoting tumor growth and metastasis. The study found that IL-6 from M2 macrophages induced METTL3-mediated m6A methylation in HCC cells, enhancing glycolysis. These findings revealed a new regulatory axis between HCC cells and the tumor microenvironment through exosomal lncRNA and provided potential therapeutic targets for HCC treatment.

## Supplementary Material

Supplementary figures and tables.

## Abbreviations

3'UTR: 3' untranslated region
ActD: actinomycin D
AREs: adenylate/uridylate-rich elements
CHOL: cholangiocarcinoma
COAD: colon adenocarcinoma
DFS: disease-free survival
ESCA: esophageal carcinoma
Exo-CM: conditional medium from exosome-exposed macrophages
HCC: hepatocellular carcinoma
HNRNPA1: heterogeneous nuclear ribonucleoprotein A1
IL-6 Abs: IL-6-neutralizing antibodies
LIHC: liver hepatocellular carcinoma
lncRNAs: long noncoding RNAs
m^6^A: N^6^-methyladenosine
METTL3: methyltransferase-like 3
OS: overall survival
TAMs: tumor-associated macrophages
TME: tumor microenvironment
