# Supplementary material for: Exosomal SLC16A1-AS1-induced M2 macrophages polarization facilitates hepatocellular carcinoma progression
Source: Int J Biol Sci. 2024 Aug 12;20(11):4341–63. doi: 10.7150/ijbs.94440 (PMC11379075; doi:10.7150/ijbs.94440)
Supplement: Supplementary file 1 — Supplementary figures and tables. [file ijbsv20p4341s1.pdf]

Exosomal lncRNA SLC16A1-AS1 promotes lactate-induced M2 polarization of macrophage to accelerate hepatocellular carcinoma progression

Supplementary Figures and Legends

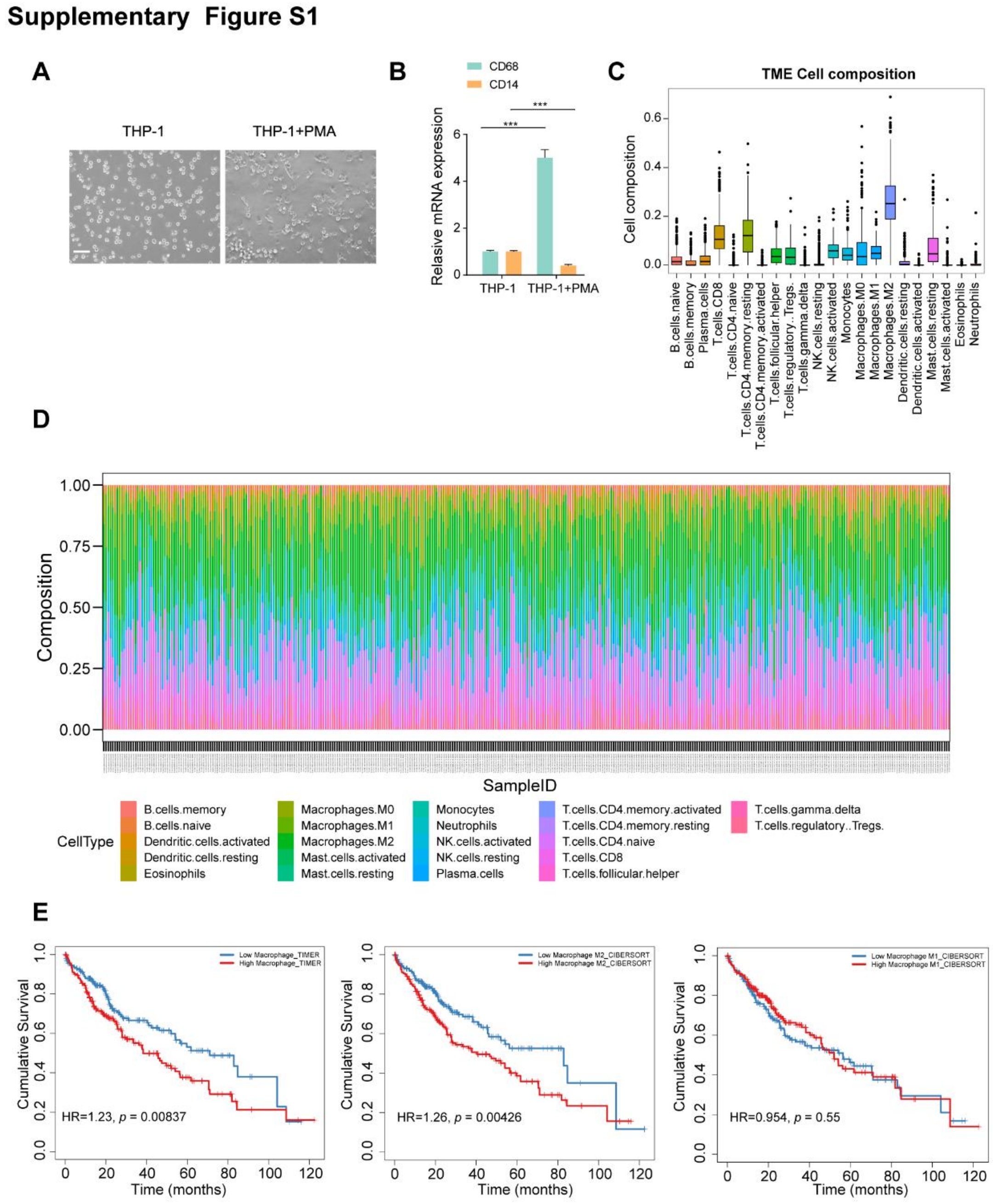

Supplementary Figure 1. High infiltration of M2 macrophages is associated with poor prognosis of HCC patients

(A) Morphological changes of THP-1 before and after PMA (100ng/ml) treatment. Scale bar:200 um. **(B)** The mRNA levels of CD68 and CD14 in THP-1 cells treated

with or without PMA. **(C)** Boxplot displaying the whole composition of immune cells in HCC. **(D)** Pile-up histogram showing the composition of immune cells in each sample. **(E)** Comparison of the percent of survival of patients with high and low infiltration of M0 macrophages, M1 macrophages or M2 macrophages using the Kaplan-Meier method.

Supplementary Figure S2

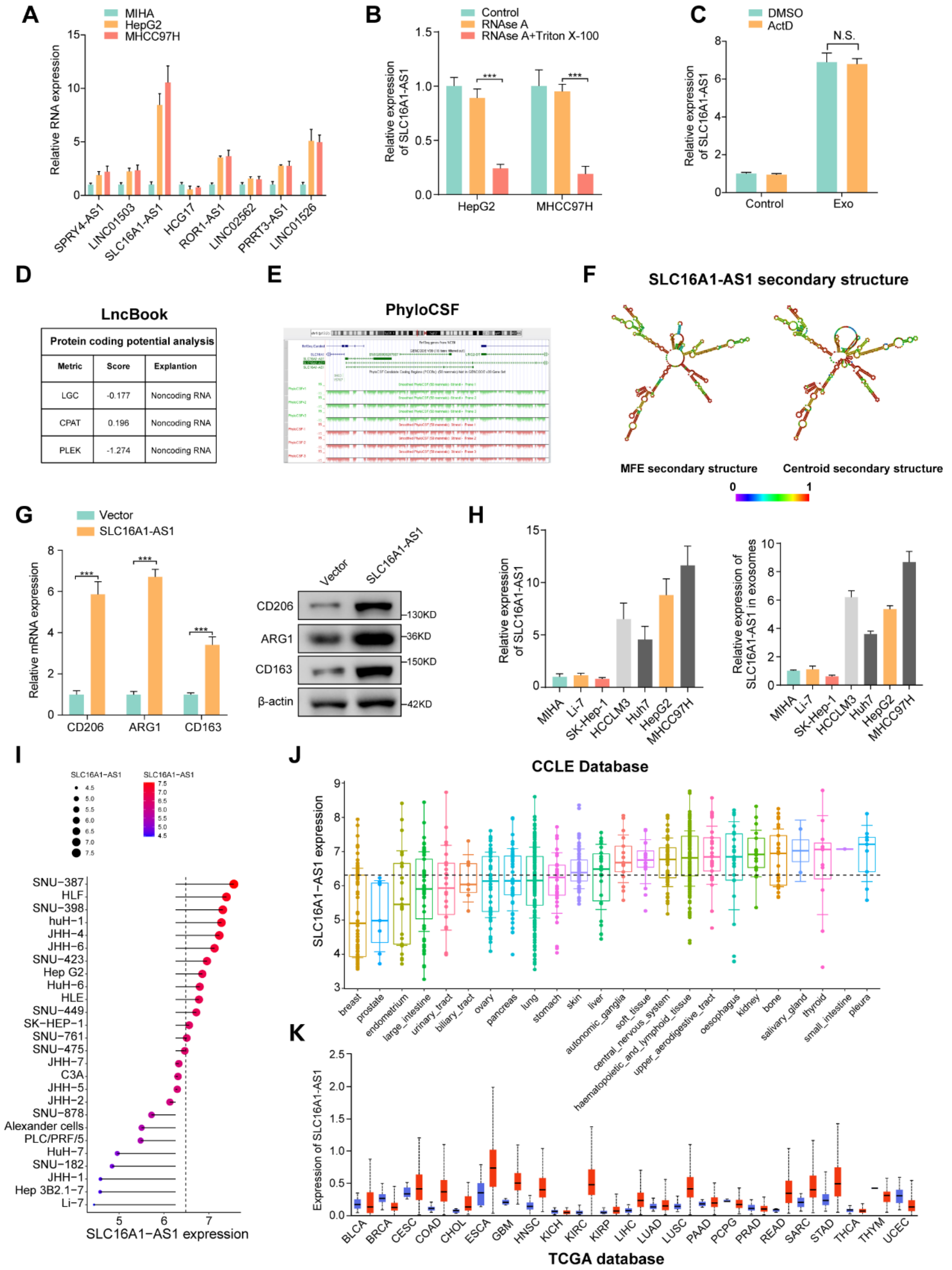

Supplementary Figure 2. The basic characteristics of SLC16A1-AS1

(A) Relative expression of lncRNAs in HCC cells and MIHA cells. **(B)** QRT-PCR analysis of SLC16A1-AS1 expression in HCC cells conditioned medium (CM) treated with RNase A (2 mg/ml) alone or combined with Triton X-100 (0.1%) for 20 min. **(C)**

The expression of SLC16A1-AS1 in exosomes incubated macrophages with or without ActD treatment was detected by qRT-PCR. **(D, E)** The coding ability of SLC16A1-AS1 was analyzed by LncBook online website and PhyloCSF. **(F)** The predicted secondary structure diagram of SLC16A1-AS1 was sourced from the online database. **(G)** M2 markers (CD206, ARG1, CD163) of macrophages after overexpression of SLC16A1-AS1. **(H)** Relative expression of SLC16AS1-AS1 in various HCC cell lines and corresponding derived exosomes was detected by qRT-PCR. **(I, J)** The expression of SLC16A1-AS1 in HCC cell lines and various cancer cell lines were obtained from the CCLE database. **(K)** The expression of SLC16A1-AS1 in cancer tissues and corresponding normal tissues was obtained from TCGA database.

Supplementary Figure S3

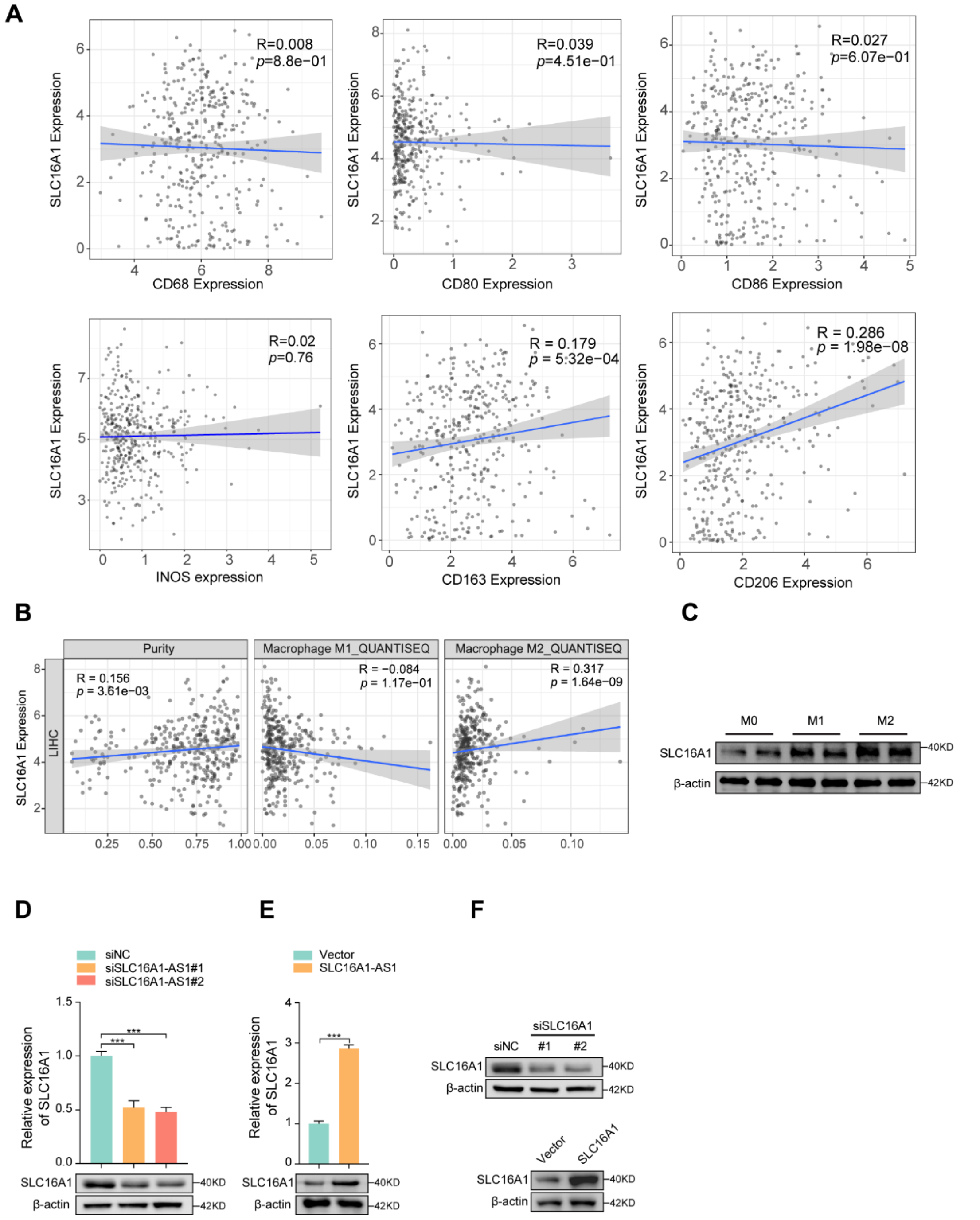

Supplementary Figure 3. Role of SLC16A1 in M2 polarization of macrophages

(A) Correlation analysis of SLC16A1 with CD68, CD86, CD80, INOS, CD163 and CD206 in HCC, respectively. **(B)** Correlation analysis between MCT1 expression and M1 and M2 macrophage infiltration in HCC. **(C)** SLC16A1 expression in THP-1 derived M0, as well as subsequently induced polarized M1 and M2 macrophages. **(D,**

E) Relative expression of SLC16A1 after overexpression or knockdown of SLC16A1-AS1 in macrophages. **(F)** Western blot analysis was used to detect the efficiency of SLC16A1 knockdown or overexpression in macrophages.

Supplementary Figure S4

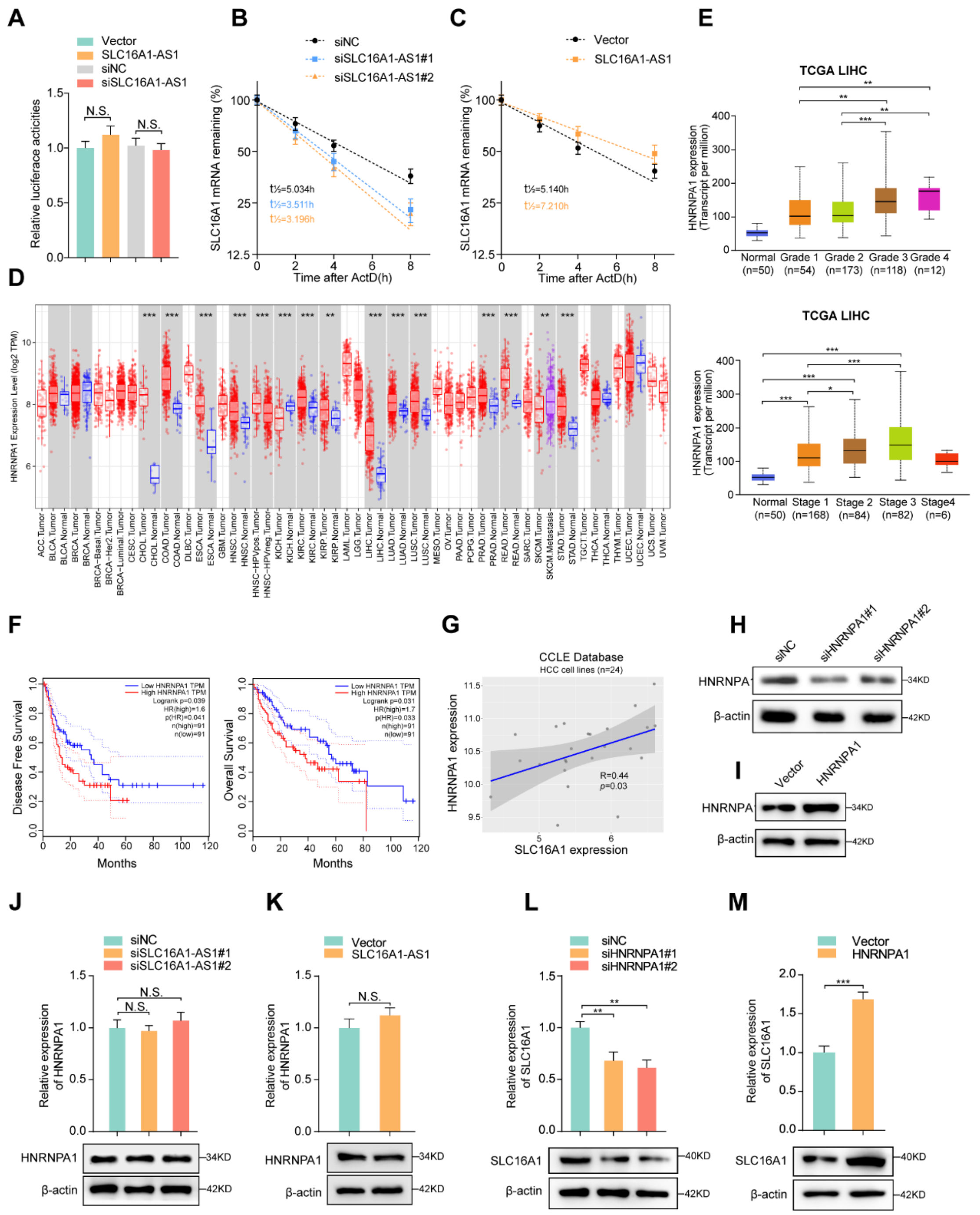

Supplementary Figure 4. HNRNPA1 is highly expressed in HCC and is associated with poor prognosis

(A) The dual-luciferase assay showing the luciferase reporter activity of the promoter region of SLC16A1 gene in macrophages transfected with vector, SLC16A1-AS1 overexpression plasmid, siNC or siSLC16A1-AS1. **(B, C)** The SLC16A1 mRNA half-life (t1/2) was detected by qRT-PCR in macrophages transfected with siNC, siSLC16A1-AS1, vector or SLC16A1-AS1 overexpression plasmid. **(D)** Bar graphs represent pan-cancer analysis of HNRNPA1. **(E)** The expression of HNRNPA1 in HCC patients with different grades (upper) or stages (lower) was obtained from TCGA database. **(F)** Kaplan-Meier analyses of disease-free survival (DFS) and overall survival (OS) in HCC patients with low and high levels of HNRNPA1 using the log-rank test. **(G)** The correlation analysis between HNRNPA1 and SLC16A1 in HCC cell lines was obtained from CCLE database. **(H, I)** Western blot was used to detect the efficiency of HNRNPA1 knockdown and overexpression in macrophages. **(J, K)** The mRNA (upper) and protein (lower) levels of HNRNPA1 in macrophages after SLC16A1-AS1 knockdown or SLC16A1-AS1 overexpression. **(L, M)** The mRNA (upper) and protein (lower) levels of SLC16A1 in macrophages after HNRNPA1 knockdown or HNRNPA1 overexpression.

Supplementary Figure S5

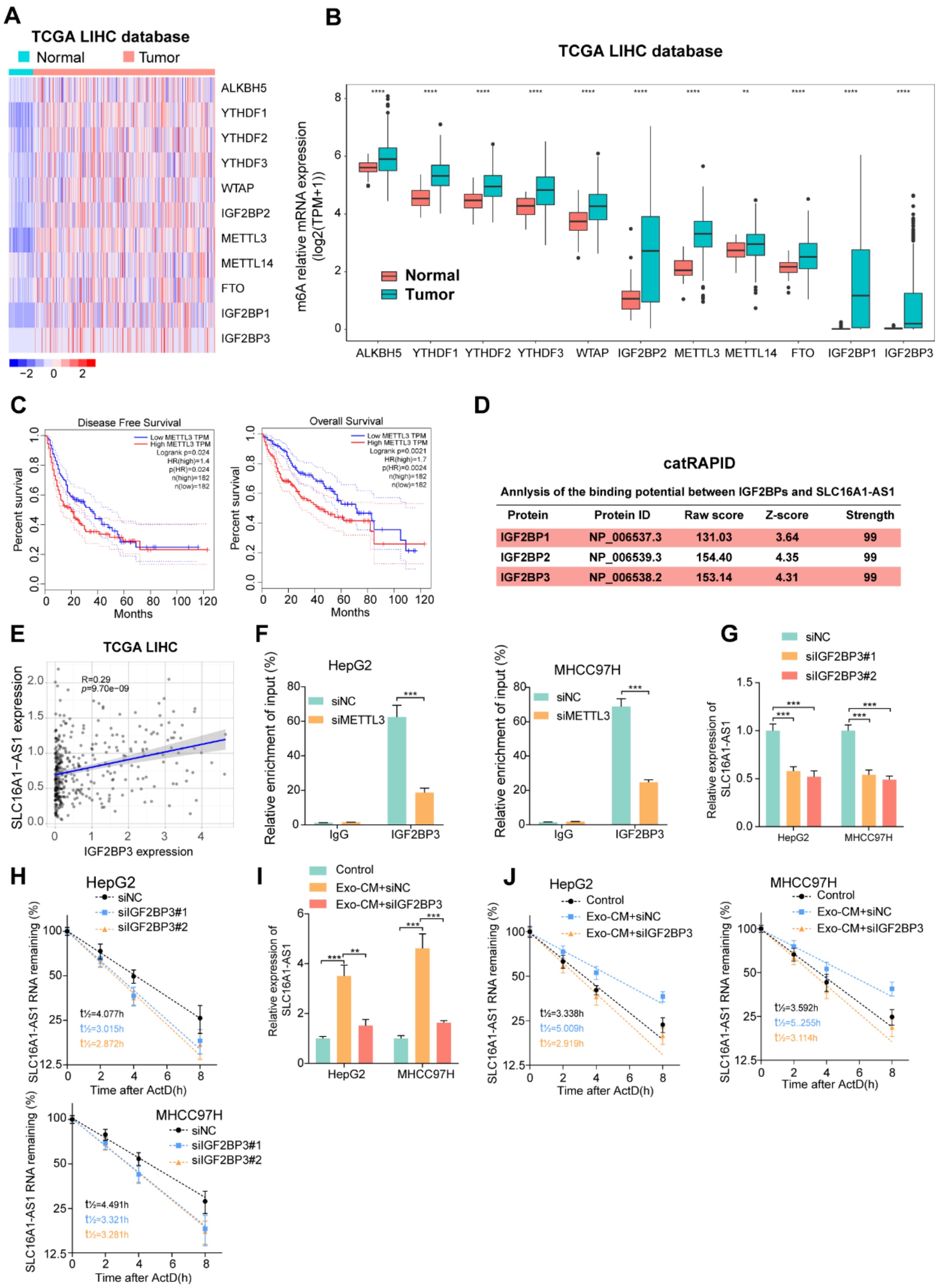

Protein	Protein ID	Raw score	Z-score	Strength
IGF2BP1	NP_006537.3	131.03	3.64	99
IGF2BP2	NP_006539.3	154.40	4.35	99
IGF2BP3	NP_006538.2	153.14	4.31	99

Supplementary Figure 5. IGF2BP3 is required for the M2 macrophages-mediated m6A modification and stability of SLC16A1-AS1 in HCC cells

(A) Heat map analysis revealed the differential expression of m^{6}A-related genes in HCC.

(B) Bar graph demonstrating the results of differential expression of m^{6}A-related genes

in HCC. **(C)** Kaplan-Meier analyses of DFS and OS in HCC patients with low and high levels of METTL3 using the log-rank test. **(D)**The binding potential of SLC16A1-AS1 and IGF2BPs family was analyzed from the *cat*RPID online website. **(E)** Correlation analysis of SLC16A1-AS1 with IGF2BP3 in HCC. **(F)** RIP assay demonstrated the enrichment of SLC16A1-AS1 on IGF2BP3 and IgG antibodies in HCC cells transfected with siNC or siMETTL3. **(G)** Relative expression of SLC16A1-AS1 after IGF2BP3 knockdown in HCC cells. **(H)** The SLC16A1-AS1 RNA half-life (t1/2) was detected by qRT-PCR in HCC cells transfected with siNC, siIGF2BP3 #1, siIGF2BP3 #2. **(I, J)** Relative expression and half-life (t1/2) of SLC161A-AS1 after knockdown of IGF2BP3 in HCC cells in the presence or absence of Exo-CM.

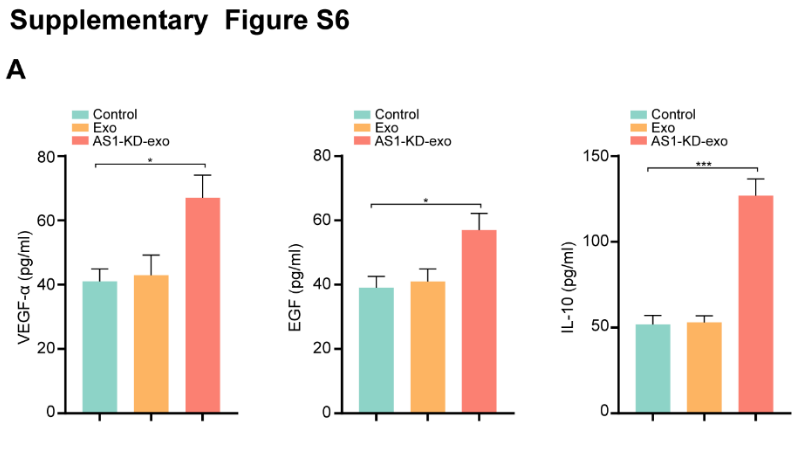

Supplementary Figure 6. Cytokine concentrations in the CM of macrophages after exosome incubation

(A) The concentrations of VEGF-α, EGF and IL-10 were detected by ELISA.

Supplementary Figure S7

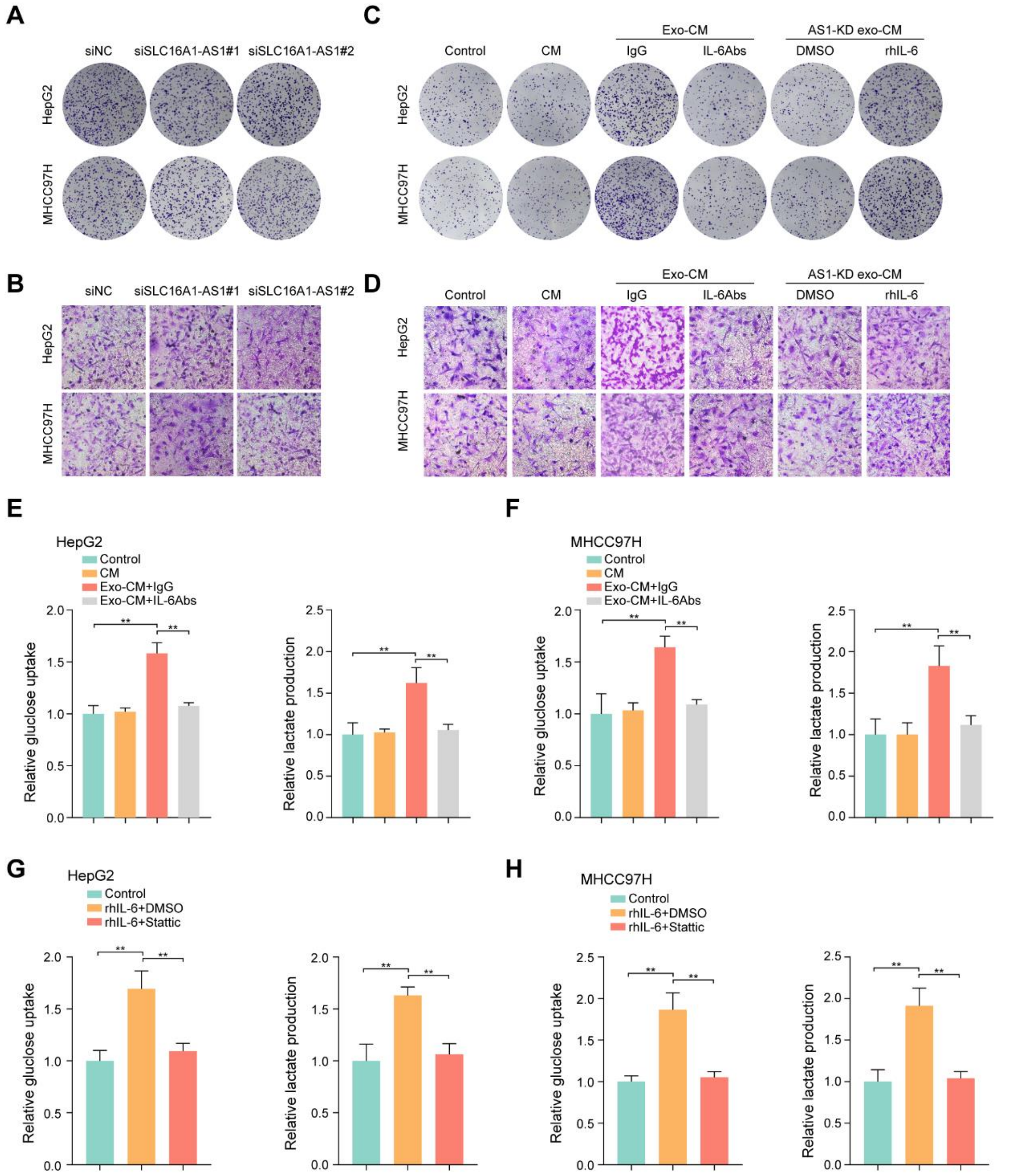

Supplementary Figure 7. Effect of macrophages on the biological effects of HCC cells

(A, B) Cell proliferation and invasion capacity of HCC cells (HepG2 and MHCC97H) with knockdown of SLC16A1-AS1. **(C, D)** Cell proliferation and invasion capacity of HCC cells (HepG2 and MHCC97H) alone or incubated with CM, Exo-CM, Exo-CM with IL-6Abs, AS1-KD-exo-CM or AS1-KD-exo-CM with rhIL-6 were determined by the colony formation and transwell coculture system, respectively. **(E, F)** Glucose uptake and lactate production of HCC cells (HepG2 and MHCC97H) alone or treated

with Exo-CM, Exo-CM+IL-6Abs. **(G, H)** Glucose uptake and lactate production of HCC cells (HepG2 and MHCC97H) alone or treated with rhIL-6, rhIL-6+Stattic.

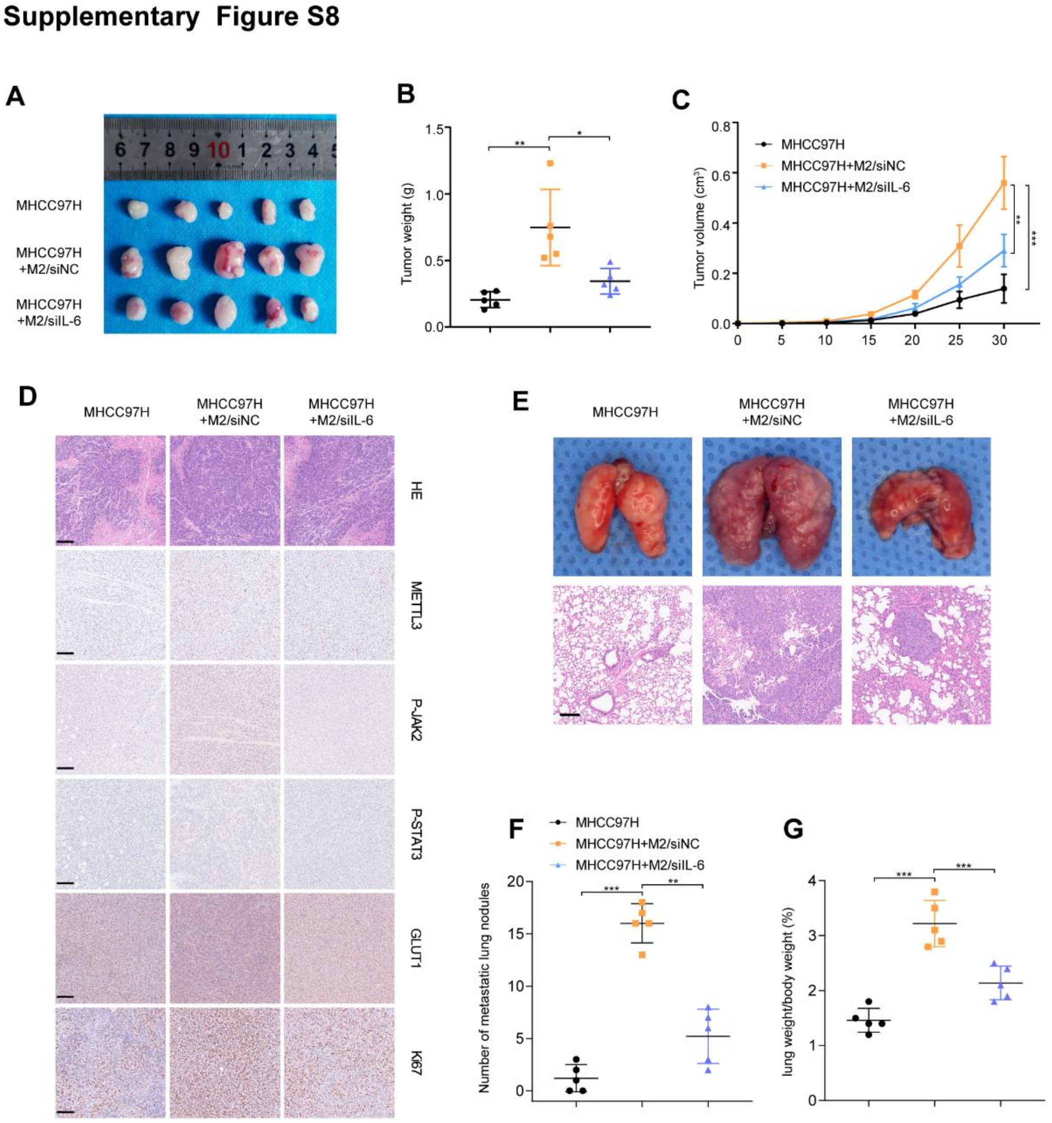

Supplementary Figure 8. Impact of M2 macrophages on the proliferation and metastasis of HCC in vivo

(A-C) The morphological characteristics of tumor xenograft, tumor weight and tumor volume in the indicated groups. (D) IHC analyzed the expression of METTL3, p-JAK2, p-STAT3, GLUT1 and Ki67 protein of tumors from the indicated groups. Scale bar: 100 μm. (E) Representative images of lung metastasis in nude mice that resulted from the indicated groups. Scale bar: 200 μm. (F) The number of metastatic nodules in each

indicated group. (G) The lung weight/body weight (%) in each indicated group.

Supplementary Figure S9

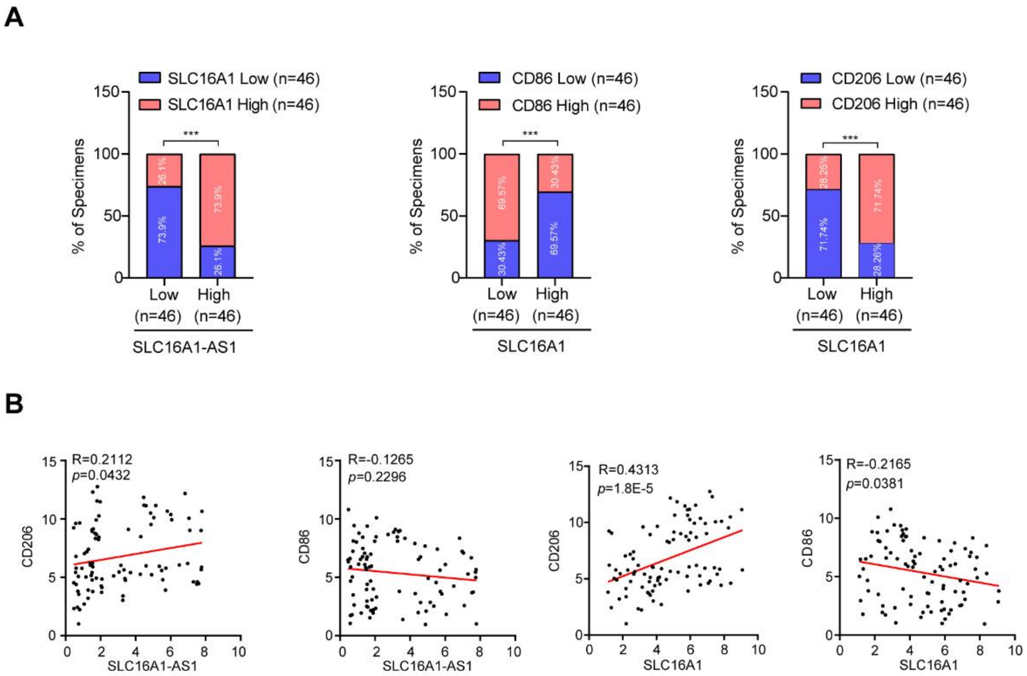

Supplementary Figure 9. SLC16A1-AS1 and SLC16A1 are highly expressed in HCC and demonstrate a strong positive correlation with M2 macrophages

(A) The proportions of SLC16A1, CD206 and CD86 in HCC specimens under corresponding groups. **(B)** Correlation analysis of SLC16A1-AS1 or SLC16A1 with CD206 and CD86, respectively.

Supplementary Methods

Cell culture

Human HCC cell lines MHCC97H, HepG2, Li7, SK-HEP-1, Huh7, HCCLM3, and normal human liver cell line MIHA were stored in our laboratory. THP-1 cell line was obtained from American Type Culture Collection (ATCC, USA). DNA fingerprints are used to confirm the exact identity of cells that grow no more than 6 months after resuscitation. Cells were cultured in Dulbecco's modified Eagle's medium (DMEM) (for the MHCC97H, HepG2, Li7, Huh7, HCCLM3 and MIHA) or RPMI-1640 medium (for the THP-1 and SK-HEP-1), supplemented with 10% FBS (Gibco, USA), 100 U/mL penicillin and 100 mg/ml streptomycin (Bio Basic Inc., Shanghai, China) in a humidified atmosphere at 37°C, 5% CO2. For macrophages maturation, THP-1 cells were treated with phorbol-12-myristate-13 acetate (PMA, 100 ng/ml, HY-18739, MCE, United States) for 24-48 hours. For polarization toward the M1 or M2 phenotype, the THP-1-derived macrophages were cultured for 96 hours in media containing lipopolysaccharide (LPS, 100 ng/ml; Solarbio, Beijing, China) or IL-4 (20 ng/mL; Peprotech, USA), respectively.

Exosomes Co-cultured with macrophages

Initially, we prepared exosomes from HCC cells that were pre-transfected with either si-SLC16A1-AS1 or si-NC. We then quantified the concentration of these exosomes utilizing Nanoparticle Tracking Analysis (NTA). Following this, we introduced the exosomes at a concentration of 100 ug/mL to the macrophages in the culture plate. This

co-culture was maintained at 37 °C for a period of 24 hours. After the co-culture period, we proceeded with the subsequent experimental assays.

Quantitative real-time PCR (qRT-PCR)

Total RNA was extracted with RNAiso Plus (TaKaRa, Kyoto, Japan). Reverse transcription and qRT-PCR were performed with PrimeScript™ RT reagent Kit (Perfect Real Time) (TaKaRa, Kyoto, Japan) according to manufacturers' protocols. Next, real-time polymerase chain reaction (PCR) amplification was performed with SYBR® Premix Ex Taq™ (Tli RNaseH Plus) (TaKaRa, Kyoto, Japan) on a StepOnePlus™ Real-time PCR system (Thermo Fisher, USA). The transcript levels were analyzed by $2^{-\Delta\Delta Ct}$ method with GAPDH as an internal control. All primers were indicated in Supplementary Table S1.

Western blot analysis

Cellular protein was extracted with 1× Cell lysis buffer for Western and IP (Beyotime, Shanghai, China). Denatured protein extracts were electrophoresed on 10% SDS-PAGE gel and transferred to an NC membrane (Millipore, USA). After blocking with 5% non-fat milk, the membranes were incubated with primary antibodies at 4 °C overnight. The HRP-conjugated secondary antibodies (Proteintech, Wuhan, China) were used to incubate the membranes for 1 h at room temperature. The membranes were washed and were detected by the chemi-luminescence method. Proteins were detected using a Bio-Rad ChemiDoc XRS+ System. Bio-Rad Image Lab software was used for densitometric analysis. The primary antibodies were listed in Supplementary Table S2.

Measurement of Glucose and Lactate

A total of 8×10^4 cells per well were seeded in 12-well plates for 12h and then changed with fresh culture medium. Cells were incubated for 24 hours after indicated treatments and collected the culture medium. The concentration of glucose and lactate in the medium with separate treatment was measured by glucose detection kit (Solarbio, Beijing, China, BC2500) and Lactic Acid assay kit (Nanjing Jiancheng Bioengineering Institute, Nanjing, China, A019-2-1) according to manufacturer's instructions. The glucose consumption and lactate production were normalized to cell numbers.

Colony Formation Assay

For transwell chamber-based cocultures, HepG2 and MHCC97H cells were planted at a density of 1000 cells in 6-well plates with conditioned medium derived from macrophages undergo indicated treatments. The cells were cocultured for 8~10 days. After clone formation, the clones were washed with 1x PBS three times, fixed with 4% paraformaldehyde for 15 mins, stained with 0.1% crystal purple for 30 mins. Finally, after washing with 1x PBS for several times, the cell colonies were counted under the microscope.

Cell transwell invasion assays

Cell invasion assays were performed using the 24-well Transwell cell culture chambers with 8-μm sized pores (Corning, Shanghai, China) which were pre-coated with or

without Matrigel (Biosciences, San Jose, USA). Then, suspension of 5×10^4 HCC cells (HepG2 and MHCC97H) in 100μl medium were added to the upper chamber, and conditioned medium derived from macrophages with indicated treatments were added to the bottom chamber. For the control, 800 μl of medium with 10% FBS was placed in the lower chamber. After incubating 24h in 37℃, 5% CO_2, the cells on the membrane of the lower chamber were fixed 4% paraformaldehyde and stained with 0.5% crystal violet, while the cells remaining in the upper chamber were removed. Five random fields (×40 magnifications) were counted under microscope.

Transfection

siRNA targeting SLC16A1, SLC16A1-AS1, IL-6, METTL3, HNRNPA1, IGF2BP3 and matched siRNA negative controls came from GenePharma (Shanghai, China). The sequences of siRNA for SLC16A1, SLC16A1-AS1, METTL3, HNRNPA1, IGF2BP3 are provided in Supplementary Table S3. Plasmids pcDNA-SLC16A1-AS1, pcDNA-LINC01549, pcDNA-LINC01693, pcDNA-SLC16A1, pcDNA-HNRNPA1 and control vectors pcDNA-3.1(Vector) were purchased from GeneChem (Shanghai, China). Opti-MEM (Gibco) and Lipofectamine 2000 (Invitrogen) were used for cell transfection according to the manufacturer's instructions.

Immunohistochemistry (IHC)

Immunohistochemical staining of HCC and paracancerous tissues with was performed at the Baiaosi Bioscience Co., Ltd., Wuhan, China. Briefly, tissues were

fixed were fixed with 4% paraformaldehyde. After paraffin embedding, the tissue paraffin blocks were processed as 4 mm sections. Following rehydration with progressively decreasing concentrations of ethanol, the specimen sections underwent high-pressure heat treatment to facilitate antigen retrieval. Subsequently, the sections were treated with 3% H_2O_2 for a duration of 10 minutes. Next, tissue sections were blocked in 5% goat serum for a period of 30 minutes and incubated overnight at 4°C with primary antibodies. The following day, slides were washed three times with PBS prior to incubation with HRP-conjugated secondary antibodies at a temperature of 37°C for 30 minutes. Slides were once again washed three times using PBS. Finally, DAB (3,3' -benzidine) was employed for staining.

Immunofluorescence (IF)

For paraffin sections of tissues, after deparaffinization and rehydration, heat-induced antigen retrieval was performed using trisEDTA buffer (pH 9.0). Tissues were stained with MCT1 antibody (Proteintech), CD206 antibody (Abmart), CD86 antibody (Proteintech) at 1:200 dilution in 5% BSA each for 1 hour at room temperature, followed by blocking with 5% bovine serum albumin (BSA). CD206/CD86 staining was pseudocolored red, and MCT1 staining was pseudocolored green. The nucleus was stained with 4',6-diamidino-2-phenylindole. The immunofluorescence images were recorded by a laser scanning confocal microscopy. Quantification of MCT1 in $CD206^+$ or $CD86^+$ cells was performed with FIJI (ImageJ) software.

Chromatin immunoprecipitation (ChIP) assay

ChIP was conducted using the EZ-ChIP™ Chromatin Immunoprecipitation Kit (Millipore, USA) according to the manufacturer's instructions. In brief, an appropriate amount of formaldehyde was added to the 10cm petri dish full of cells to a final concentration of 1% and incubated at 37°C for 15 minutes to cross-link the target protein and the corresponding genomic DNA. Sonicate the collected cells to break most of the DNA into 200-1000bp. 20ul samples were taken as input for subsequent detection. The rest of the samples were incubated with anti-STAT3 antibody or negative control antibody IgG at 4 °C overnight. Protein G agarose beads were added to precipitate the protein-DNA complex. After washing the complex, the protein was digested with protease K, and the remaining supernatant was purified. Enriched DNA fragments were amplified by PCR reaction and analyzed by 2% agarose gel electrophoresis. Primers for ChIP-qPCR are listed in in Supplementary Table S4.

Quantification of cytokines by enzyme-linked immunosorbent assay (ELISA)

The concentrations of IL-6 (D6050), VEGF-α (DVE00), EGF (DEG00), IL-10 (D1000B) cytokines were estimated for each experimental condition by ELISA, using commercial kits purchased from R&D Systems (Minneapolis, MN, USA), according to the manufacturer's instructions. In brief, the reaction wells were closed with 5% fetal bovine serum for 40 min. Then, the blank, negative, and positive control wells were prepared in the reaction well, and 100 µl freshly diluted enzyme labeled secondary

antibody was added for incubation at 37°C for 60 min and washed. Each reaction well was added with temporarily prepared TMB substrate solution at 37°C for 5-10 min, and 50 µl stop buffer was added to terminate the development. Finally, the absorbance was measured at 450nm.

RNA- Fluorescence in situ hybridization (RNA- FISH)

The FISH Tag™ RNA Multicolor Kit (Invitrogen, USA) and MAXIscript® Kit (Ambion, USA) was used for single molecule RNA FISH based on manufacturer's instructions. In brief, SLC16A1-AS1 probes were transcribed *in vitro* with the T7 RNA polymerase and labeled with red fluorescence. Finally, labeled probes were hybridized overnight with HCC cell samples at 55 °C in a humid environment. 18 S and U6 were used as the cytoplasmic and nuclear markers, respectively. Stained results were observed by using LSM 5 Pascal Laser Scanning Microscope (Carl Zeiss, Germany). Formaldehyde and TritonX-100 were used to fix and permeabilize cells separately. Probe sequences were given in Supplementary Table S4.

RNA stability

To measure RNA stability of SLC16A1 and SLC16A1-AS1 in HepG2 and MHCC97H cells, 5 µg/ml actinomycin D (Act-D, Catalogue #A9415, Sigma, St. Louis, MO, USA) was added to cells. After incubation at the 2, 4, or 8 h, cells were collected, and RNA was isolated for qRT-PCR. The RNA half-life (t1/2) was calculated by ln2/slope, and GAPDH was used for normalization.

Cytoplasmic and nuclear RNA isolation

Cytoplasmic and nuclear RNA was extracted using Thermo Fisher BioReagents (Thermo Fisher Scientific, USA) according to the manufacturer's instructions. QRT-PCR analysis was performed using SYBR® Green Master Mix (Invitrogen, New York, USA) to amplify the localization of SLC16A1-AS1 assay.

Luciferase reporter assay

For the dual-luciferase reporter assay, a 2000-bp DNA fragment upstream from the METTL3, SLC16A1-AS1 or SLC16A1 promoter was cloned into pGL3-Basic plasmid (Promega, USA). The reporter activity was measured by using a luciferase assay kit (Promega, USA) and plotted after normalizing with respect to Renilla luciferase activity. Firefly luciferase activity was measured by the Dual Luciferase reporter assay system (Promega, Madison, WI, USA), and normalized by Renilla luciferase activity. All experiments were repeated three times independently with five samples per group.

MeRIP

Total RNA was isolated from HepG2 and MHCC97H cells incubated with M0-CM or M0-Exo-CM by Trizol extraction respectively. In total, 5μg of anti-m^6A antibody (Abcam, USA, ab190886) was pre-bound to Protein G magnetic beads in immunoprecipitation buffer for 2h. Protein G beads were then added and incubated at 4 °C for 2 h. Samples were washed five times with immunoprecipitation buffer, and

RNA was eluted from the beads by incubating with 400μl of 0.5 mg/ml m6ATP for 1 h at 4°C. Following ethanol recipitation, the input RNA and eluted RNA were reverse transcribed with random hexamers and enrichment was determined by qRT–PCR.

Flow cytometry

Macrophages were processed into single cell suspensions, and then incubated with CD206-APC (human) antibodies (Biolegend, USA) for 1 h at 4 °C. The cells were then washed twice with 4 ml of flow buffer, then centrifuged, and resuspended in 0.5 ml of flow buffer for analysis. All the tests were controlled by the homologous isotype control antibodies. Flow cytometry was performed using a FACSC Calibur flow cytometer (BD Biosciences, USA). Flow cytometric analysis was performed on FlowJo software (FlowJo, USA)

Supplementary Tables

Supplementary Table S1. The sequences of PCR primers.

Primer	Sequence
SLC16A1-AS1	Forward: 5’- CGACAGTAGTAGAACCGACCT-3’
	Reverse: 5’- GCAGATTACCATAGCAGCATC-3’
SPRY4-AS1	Forward: 5’- ACCAGTTAAGAGCCTGCCAC-3’
	Reverse: 5’- TCTATGCAGCCTCCCATTGC-3’
LINC01503	Forward: 5’-CCCTGATAGATGGGGTGCG-3’
	Reverse: 5’- ACACCAAGACAAAGGCCTGC-3’
HCG17	Forward: 5’-CTTGCAAATAGGCCACACCG-3’
	Reverse: 5’- GAGGAACAGCCGTGTCATCA-3’
ROR1-AS1	Forward: 5’-TGAAACTTCCTGGCCTTGGTT-3’
	Reverse: 5’- GCATGCAGTTTCACAAGTCGT-3’
LINC02562	Forward: 5’-AGTTCAAGTATGTGGCTCCTGA-3’
	Reverse: 5’- TGGACTTGGTTTGCTAAGTTGA-3’
PRRT3-AS1	Forward: 5’-GAAATGTTTAGCCCGGTGCC-3’
	Reverse: 5’- GTCCTACTTTCAGAGGGGCG-3’
LINC01526	Forward: 5’-CCTTGAGACTTTGCAGTGTGC-3’
	Reverse: 5’- AGGAGCAAACAAAGGGCAGG-3’
SLC16A1	Forward: 5’-CACCCTAAACAAGAGAAACG-3’
	Reverse: 5’-CAAAAGCCAGAATGGAAAGA-3’
CD206	Forward: 5’-GGGTTGCTATCACTCTCTATGC-3’
	Reverse: 5’-TTTCTTGTCTGTTGCCGTAGTT-3’
CD163	Forward: 5’-TTTGTCAACTTGAGTCCCTTCAC-3’
	Reverse: 5’-TCCCGCTACACTTGTTTTCAC-3’
ARG1	Forward: 5’-GTGGAAACTTGCATGGACAAC-3’
	Reverse: 5’-AATCCTGGCACATCGGGAATC-3’
CD86	Forward: 5’-CTGCTCATCTATACACGGTTACC-3’
	Reverse: 5’-GGAAACGTCGTACAGTTCTGTG-3’
CD68	Forward: 5’-CTTCTCTCATTCCCCTATGGACA-3’
	Reverse: 5’-GAAGGACACATTGTACTCCACC-3’
CD14	Forward: 5’-GACCTAAAGATAACCGGCACC-3’
	Reverse: 5’-GCAATGCTCAGTACCTTGAGG-3’
iNOS	Forward: 5’-AGGGACAAGCCTACCCCTC-3’
	Reverse: 5’-CTCATCTCCCGTCAGTTGGT-3’
IL-6	Forward: 5’-AATAACCACCCCTGACCCAAC-3’
	Reverse: 5’-ACATTTGCCGAAGAGCCCT-3’
TNF-α	Forward: 5’-CCTCTCTCTAATCAGCCCTCTG-3’
	Reverse: 5’-GAGGACCTGGGAGTAGATGAG-3’
IL-12	Forward: 5’-CCTTGCACTTCTGAAGAGATTGA-3’
	Reverse: 5’-ACAGGGCCATCATAAAAGAGGT-3’
IL-18	Forward: 5’-GCTGAAGATGATGAAAACCTGGA-3’

	Reverse: 5’-GAGGCCGATTTCCTTGGTCA-3’
CXCL9	Forward: 5’-CCAGTAGTGAGAAAGGGTCGC-3’
	Reverse: 5’-AGGGCTTGGGGCAAATTGTT-3’
VEGF-α	Forward: 5’-AGGGCAGAATCATCACGAAGT-3’
	Reverse: 5’-AGGGTCTCGATTGGATGGCA-3’
EGF	Forward: 5’-TGTCCACGCAATGTGTCTGAA-3’
	Reverse: 5’-CATTATCGGGTGAGGAACAACC-3’
IL-10	Forward: 5’-GACTTTAAGGGTTACCTGGGTTG-3’
	Reverse: 5’-TCACATGCGCCTTGATGTCTG-3’
HNRNPA1	Forward: 5’-TGCTATGCGGGTAGGTAAGA-3’
	Reverse: 5’-GGAAATGGGTCAAAGGAAGA-3’
METTL3	Forward: 5’-TTGTCTCCAACCTTCCGTAGT-3’
	Reverse: 5’-CCAGATCAGAGAGGTGGTGTAG-3’
METTL14	Forward: 5’-AGTGCCGACAGCATTGGTG-3’
	Reverse: 5’-GGAGCAGAGGTATCATAGGAAGC-3’
FTO	Forward: 5’-ACTTGGCTCCCTTATCTGACC-3’
	Reverse: 5’-TGTGCAGTGTGAGAAAGGCTT-3’
WTAP	Forward: 5’-CTTCCCAAGAAGGTTCGATTGA-3’
	Reverse: 5’-TCAGACTCTCTTAGGCCAGTTAC-3’
ALKBH5	Forward: 5’-CGGCGAAGGCTACACTTACG-3’
	Reverse: 5’-CCACCAGCTTTTGGATCACCA-3’
GAPDH	Forward: 5’-TGAACGGGAAGCTCACTGG-3’
	Reverse: 5’-TCCACCACCCTGTTGCTGTA-3’
β-actin	Forward: 5’-ACTTAGTTGCGTTACACCCTT-3’
	Reverse: 5’-GTCACCTTCACCGTTCCA-3’

Supplementary Table S2. The antibodies used in this article.

ANTIBODIES	SOURCE	IDENTIFIER
SLC16A1	Proteintech	20139-1-AP
HNRNPA1	Proteintech	11176-1-AP
CD206	Abmart	T40027
ARG1	Proteintech	66129-1-Ig
CD86	Proteintech	13395-1-AP
INOS	Proteintech	18985-1-AP
CD63	Proteintech	25682-1-AP
CD68	Proteintech	28058-1-AP
Alix	Proteintech	12422-1-AP
p-JAK2	ABclonal	AP0531
JAK2	Abmart	T55287
STAT3	Proteintech	60199-1-Ig
p-STAT3	ABclonal	AP0070
p-c-Raf	ABclonal	AP0498
c-Raf	Proteintech	66592-1-Ig

p-ERK1/2	ABclonal	AP0472
ERK1/2	Proteintech	11257-1-AP
p-AKT	ABclonal	AP0140
AKT	Proteintech	60203-2-Ig
p-STAT6	ABclonal	AP0456
STAT6	Proteintech	66717-1-Ig
METTL3	Proteintech	15073-1-AP
IGF2BP3	Proteintech	14642-1-AP
β-actin	Proteintech	66009-1-Ig

Supplementary Table S3. The sequence of siRNA

siRNA	Sequence (5′-3′)
SLC16A1-AS1-siRNA#1 sense	GCCGGACUGUUGUUUGCAATT
SLC16A1-AS1-siRNA#2 sense	GGUUAAGUGAGCAAAUCUUTT
SLC16A1-siRNA#1 sense	GGAAGAGACCAGUAUAGAUTT
SLC16A1-siRNA#2 sense	GCUUCAUUGUUACAUGUAATT
IL-6-siRNA#1 sense	CUCAGAUUGUUGUUGUUAAUG
IL-6-siRNA#2 sense	GAACUUAUGUUGUUCUCUAUG
HNRNPA1-siRNA#1 sense	GCACGCUCCAUGAAAGCUATT
HNRNPA1-siRNA#2 sense	GAGCUGUUCUGCAGCUCAATT
METTL3-siRNA#1 sense	GCAAGUAUGUUCACUAUGATT
METTL3-siRNA#2 sense	CAGUGGAUCUGUUGUGAUATT
IGF2BP3-siRNA#1 sense	GCUACAGCAUGCAGAGCAATT
IGF2BP3-siRNA#2 sense	CAAAGAUAAUCAUCAUCAATT
NC-siRNA sense	UUCUCCGAACGUGUCACGUUU

Supplementary Table S4. Primers used for ChIP/Pull down/FISH

	**Sequence**
CHIP:METTL3	S1: F 5'-AAACTGGCTCACAGCTCCTT-3'<br>R 5'-GTCTTGAACTCCTGACCTTAG-3'<br>S2: F 5'-CTTTAACACAAGTCAACTCTCAGG-3'<br>R 5'-GCAAGACAGAAAAGTCATTTCC-3'
Pulldown: SLC16A1-AS1	Full length<br>F: 5'-TAATACGACTCACTATAGGGGCTCTAGGCAATCT-3'<br>R: 5'-GCATCCAGTCTATCAACCTGCTCTTCTTT-3'<br>T1 F: 5'-TAATACGACTCACTATAGGGGCGCGCTGCGCCCCTGCTGAGC-3'<br>R: 5'-CGGCTCTACAGCGCCGGCCCCCAG-3'<br>T2 F: 5'-TAATACGACTCACTATAGGGGGCATGGGCTGGGATGTGTTTGGA-3'<br>R: 5'-TTCCACGTGGCCGAGGTGCACACC-3'<br>T3 F: 5'-TAATACGACTCACTATAGGGAAGCGAGAATGCAGAGGCCGGACG-3'<br>R: 5'-GATCACTTCAACACACACGTTGGT-3'

	T4 F: 5'-TAATACGACTCACTATAGGGAAATAACCATGGAGTGTCTTTGAT-3'<br>R: 5'-AATCTCAGGTTCTTCATTTGCAAA-3'<br>T5 F: 5'-TAATACGACTCACTATAGGGGAAGAAGAGTAGAGATTGGAGGAT-3'<br>R: 5'-TGTTACACTTTAAGTTTTAGGGTA-3'
	3' UTR F: 5'-TAATACGACTCACTATAGGGATCCATGGGGCTGAAGGGTAAAT-3'<br>R: 5'-TTTATACTTGCTTTACTTTTATTTTTTC-3'<br>3' UTR-ARE1 F: 5'-TAATACGACTCACTATAGGGTTTTAATAGTGTCATAAAGATTAT-3'<br>R: 5'-GAAAATGTCTGAAATATATTAC-3'<br>3' UTR-ARE2 F: 5'-TAATACGACTCACTATAGGGCTTGCTTCATTGTTACATGT-3'<br>R:5'-TATAGCTAGCAGAAACATATGC-3'<br>3' UTR-ARE3 F:5'-TAATACGACTCACTATAGGGTAAGCATCCCAAGAAAAGGTA-3'<br>R:5'-TAAGCATCCCAAGAAAAGGTA-3'<br>3' UTR-ARE4 F:5'-TAATACGACTCACTATAGGGATCAGCTGAATTCACTTAAGTT-3'<br>R:5'-CTATCTTTATTCGTTTGCCTG-3'<br>3' UTR-ARE5 F:5'-TAATACGACTCACTATAGGGCTTACCTGAAAAGTAGAGAAAT-3'<br>R:5'-TGAAAATGAAGACACAGCAATA-3'<br>3' UTR-ARE6 F:5'-TAATACGACTCACTATAGGGCTTGAGGAAAATATTGCTGTGT-3'<br>R:5'-CTTAAGGTTAACTGCTCTGGAT-3'
SLC16A1-AS1probe for FISH	F: 5'-TAATACGACTCACTATAGGGATTTTATTATTGTT-3'<br>R: 5'-TCGGTCTCACCCCTCAGTTTCT-3'

Supplementary Table S5. The mass spectrometry identification of proteins list

Accession	Gene Symbol	Score AS	Coverage AS	# Peptides AS	# PSM AS	Score S	Coverage S	# Peptides S	# PSM S
P80404	ABAT	62.06	2.40	1	1				
L0R4T3	ABCF2	38.69	13.43	1	1				
O75944	ACON	135.95	7.33	4	4	84.39	3.67	2	2
A0A2R8YFE2	ACTB	310.66	50.63	3	10	242.68	39.24	3	8
Q562R1	ACTBL2	345.51	14.10	4	11	518.56	8.24	3	14
O43707	ACTN4	69.56	4.72	4	4				
C9JKR2	ALB	49.40	5.04	2	2	44.78	5.04	2	2
H3BUH7	ALDOA	147.54	29.03	5	5	60.24	5.81	1	1
Q5T3N1	ANXA1	37.88	5.39	1	1				
H0YNA	ANXA2	95.77	48.10	3	3				

0									
H0YJ08	AP1G2	0.00	16.78	1	1				
K7EJ01	AP2B1	35.20	7.32	1	1				
P61204	ARF3	101.11	22.10	3	4	51.90	6.08	1	1
M0R116	ATP1A3	244.99	14.85	10	11	154.21	5.39	3	3
Q0QEN7	ATP5B	223.64	21.57	6	9	60.55	6.07	2	2
K7EK77	ATP5F1A	160.76	20.77	3	3	61.23	5.80	1	1
P38606	ATP6V1A	42.37	3.89	2	2				
H0YC04	ATP6V1B2	65.85	11.06	2	2				
Q9BRL5	CALM3	43.19	10.88	2	2	45.53	5.44	1	1
K7EJB9	CALR	63.09	12.55	2	2				
Q8IWE0	CAMK2A	61.01	5.23	2	2	46.82	3.97	1	1
P27824	CANX	53.65	1.52	1	1				
Q7L4N0	CAPZB	28.25	12.50	1	1				
E9PLJ3	CFL1	45.15	10.13	1	1				
P12277	CKB	51.38	11.81	3	3	94.33	6.30	2	2
C9J8P9	CLTA	33.29	4.08	1	1				
Q00610	CLTC	105.41	2.81	4	4	50.10	0.72	1	1
A0A024R1T5	CNP	35.59	3.24	1	1				
P02458	COL2A1					17.08	1.21	1	1
D3DPU8	COL9A2					0.00	7.06	1	1
F8W642	CS	82.26	24.24	1	1				
A0A0S2Z3L4	CTSD	55.38	9.64	1	1				
P81605	DCD	56.84	20.00	2	2	46.24	10.00	1	1
Q6MZV7	DKFZp686C11235	0.00	8.03	1	1				
A0A1B0GUX5	DNM1	106.97	11.88	4	4	51.29	3.13	1	1
A0A0D9SFH5	DOCK9					0.00	4.82	1	3

Q16555	DPYSL2	99.48	14.16	6	6	72.50	4.20	2	2
Q9HB00	DSC1	0.00	1.79	1	1				
Q02413	DSG1	66.30	2.86	2	2	38.93	1.33	1	1
P15924	DSP	45.27	0.31	1	1	49.00	0.24	1	1
Q96RE1	EEF1A1L14	86.48	10.55	4	6	33.06	3.02	1	1
E7EQG2	EIF4A2	60.25	6.63	2	2	49.67	2.76	1	1
A0A2R8Y6G6	ENO1	243.20	9.45	3	4	142.42	9.91	3	4
F5H0C8	ENO2	276.42	27.30	6	8	80.29	5.71	1	2
Q5D862	FLG2	70.93	0.46	1	1	52.89	0.46	1	1
P04406	GAPDH	94.19	14.63	3	3				
P15104	GLUL	91.91	7.51	2	2				
A0A1W2PP87	GNAO1	41.30	10.53	1	1				
B1AKQ8	GNB1	69.83	19.63	2	2	94.51	19.63	2	2
P51674	GPM6A	28.83	8.99	2	2				
B3KTX1	GSDMDC1					40.73	14.09	1	1
H0YFX9	H2AJ	0.00	20.65	1	1	45.68	7.61	1	1
P62805	H4-16; H4C2; H4C11; H4C14; H4C9; H4C5; H4C12; H4C8; H4C13; H4C1; H4C3; H4C4; H4C6; H4C15	70.65	9.71	1	1	77.73	21.36	2	2
Q5XTR9	HBD/HBB	31.80	26.47	1	1				
V9HWC6	HEL-S-39	32.71	6.25	1	1				
A0A0K	HEL-S-					46.74	10.00	1	1

0K1J6	96								
F8VTQ5	HNRNPA1					138.95	11.03	1	1
I6L957	HNRNPA2B1	71.23	7.63	2	2	68.69	4.02	1	1
Q5T6W2	HNRNPK	110.72	5.54	2	3				
O75544	HPCAL1	44.28	7.27	1	1				
Q9BS19	HPX	0.00	8.27	1	1				
Q86YZ3	HRNR	58.56	5.26	4	4	35.12	0.77	1	1
P07900	HSP90AA1	249.48	9.84	6	7	149.44	5.74	3	4
P11021	HSPA5	151.84	10.86	6	6	69.31	5.81	3	3
E9PKE3	HSPA8	196.53	14.99	8	8	90.84	6.22	3	3
H0YMU3	IDH3A	55.86	5.71	1	1				
H7C1W2	IDH3G	47.05	4.02	1	1				
A0A2U8J8J8	IgH	44.78	22.47	2	2	37.64	16.85	1	1
P01859	IGHG2	78.25	12.27	4	5	42.93	5.21	1	1
P01861	IGHG4					17.59	10.09	1	1
A0A449C189	IGHV5-51	51.10	12.24	1	1				
A0A075B6S2	IGKV2D-29	139.04	10.83	1	5	203.42	16.67	2	7
Q16352	INA	117.57	6.01	3	3				
H0YBM2	IRGM	0.00	11.27	1	1				
Q5T749	KPRP	37.32	1.38	1	1				
H6VRF8	KRT1	1881.41	51.71	25	64	1422.28	29.66	18	41
P13645	KRT10	1709.13	50.00	24	63	1003.49	39.73	17	31
A0A024R1X6	KRT14	390.84	24.52	7	11	181.06	15.33	4	5
P02533	KRT14	610.80	22.88	10	19	349.93	11.86	6	10
P19012	KRT15	170.80	5.48	3	6	106.03	5.48	3	3

P08779	KRT16	378.60	15.86	7	15	312.72	11.84	6	9
Q04695	KRT17	242.45	8.80	4	7	115.24	6.25	3	3
I6L965	KRT18	205.54	9.95	4	8	355.94	4.30	2	11
P35908	KRT2	1308.88	42.10	24	47	837.97	33.02	18	25
P13647	KRT5	461.90	22.37	13	18	249.60	15.59	9	9
Q0IIN1	KRT77	231.16	8.65	5	9	121.61	2.08	1	3
Q969I0	KRT8	452.53	19.45	7	20	169.59	5.48	2	5
P35527	KRT9	973.06	43.82	19	37	828.53	33.07	17	27
P00338	LDHA	159.44	13.86	4	4				
P07195	LDHB	54.12	7.49	2	2	43.14	4.49	1	1
F8VV32	LYZ	35.99	8.65	1	1				
E9PMR5	MBP	65.81	34.69	3	3	53.21	22.45	2	2
B9A041	MDH1	90.24	9.52	2	2				
Q0QF37	MDH2	49.72	7.21	2	2	35.92	3.61	1	1
H0YI43	MYL6	48.81	30.12	2	2				
B3KM80	NCL	89.12	5.78	3	3	58.07	1.49	1	1
O00483	NDUFA4	0.00	12.35	1	1				
E5RI98	NPM1	0.00	18.92	1	1				
I3L2G1	NSF	76.01	9.56	1	1				
K7ELW0	PARK7	45.45	8.88	1	1				
H3BSS4	PCBP2	0.00	5.98	1	1				
H3BTN5	PKM	206.36	22.27	7	8	101.34	9.69	4	4
E9PPC8	PPP3CA	49.82	6.06	1	1				
F6U1T9	PPP3R1	31.90	10.00	1	1				
A0A0A0MRQ5	PRDX1	66.22	19.59	2	2	49.78	11.34	1	1
Q45KI0	PRSS1					52.33	23.81	1	1
Q2NLD4	PURA	52.11	3.16	1	1				
P11216	PYGB	24.09	1.42	1	1				
P20336	RAB3A	88.27	15.91	3	3	56.72	3.64	1	1

C9J7D1	RAB7A	36.95	12.09	1	1				
B1AH78	RAC2	41.06	12.65	2	2				
B4DV51	RAN	41.04	7.81	1	1				
H7C2U2	RPL10	23.89	28.57	1	1				
Q5VVC8	RPL11	23.49	8.38	1	1				
A0PJ62	RPL14	86.00	9.68	1	2				
E7EX53	RPL15	51.52	6.77	1	1				
F8VUA6	RPL18					32.27	10.00	1	1
C9JD32	RPL23					24.20	21.98	1	1
G3V210	RPLP0	55.50	13.25	2	2				
P05387	RPLP2	97.72	18.26	2	2				
M0QX76	RPS16	37.76	20.00	1	1				
E9PQ96	RPS3	39.66	9.89	1	1				
D6RGE0	RPS3A					89.93	26.15	1	2
M0QZN2	RPS5	0.00	11.19	1	1				
P31151	S100A7					62.66	11.88	1	1
A0A0C4DFV9	SET	0.00	6.77	1	1				
A0A024R0G8	SIRT2	52.68	1.99	1	1				
A0A0S2Z3C4	SLC25A4	51.28	10.80	2	2				
P05141	SLC25A5	79.66	7.38	2	2				
A0A0A0MSS0	SNAP25	58.51	24.73	2	2				
H6UYS7	SNCA	65.19	20.41	1	1	55.72	20.41	1	1
J3QLE5	SNRPN	56.40	4.73	1	1				
P61266	STX1B	61.89	5.56	2	2				
A0A1B0GW76	STXBP1	32.48	3.33	1	1				
A0A0U1RQF8	SUCLA2	50.08	5.24	1	1				
A0A087WW91	SYN2	102.52	7.24	1	1				

H7C4W3	SYP	34.08	4.93	1	1	55.55	3.94	1	1
A4FU00	SYT2	62.25	3.79	1	1	51.54	3.79	1	1
H0YKX5	TPM1	38.99	7.04	1	1	55.87	7.04	1	1
A0A087WWU8	TPM3	61.98	9.69	2	2	55.87	4.41	1	1
O94811	TPPP	30.97	5.94	1	1				
Q5CAQ5	TRA1	50.39	2.74	2	2				
Q71U36	TUBA1A	292.12	32.59	10	16	211.30	15.74	5	8
P68366	TUBA4A	178.31	28.57	8	10	80.39	8.26	2	2
P68371	TUBB4B	481.10	19.78	7	21	211.50	23.37	7	11
P10599	TXN	43.40	12.38	1	1	39.55	8.57	1	1
F5H265	UBC	34.18	12.08	1	2				
P31930	UQCRC1	65.95	1.88	1	1				
K7ENK9	VAMP2	62.12	10.29	1	1				
C9JUP7	VCP	50.04	6.96	1	1				
B3KTS5	VDAC1	84.72	7.42	2	2	58.90	3.89	1	1
B4DKM5	VDAC2	107.88	8.63	2	2				
P61981	YWHAG	114.50	27.13	5	6	63.35	4.05	1	1
Q9H4N8	YWHAH	84.61	11.36	2	2	63.35	5.68	1	1
P63104	YWHAZ	133.12	32.24	6	7	110.74	8.98	2	2
